# Unveiling the Metabolic Changes on Muscle Cell Metabolism Underlying *p*-Phenylenediamine Toxicity

**DOI:** 10.3389/fmolb.2017.00008

**Published:** 2017-03-06

**Authors:** Igor Marín de Mas, Silvia Marín, Gisela Pachón, Juan C. Rodríguez-Prados, Pedro Vizán, Josep J. Centelles, Romà Tauler, Amaya Azqueta, Vitaly Selivanov, Adela López de Ceraín, Marta Cascante

**Affiliations:** ^1^Departament de Bioquímica i Biologia Molecular, Facultat de Biología, Universitat de BarcelonaBarcelona, Spain; ^2^Department of Environmental Chemistry, Institute of Environmental Assessment and Water Research, Consejo Superior de Investigaciones CientíficasBarcelona, Spain; ^3^Departamento de Farmacología y Toxicología, Facultad de Farmacia y Nutrición, Universidad de NavarraPamplona, Spain

**Keywords:** tracer-based metabolic data, metabolic modeling, metabolic pathways, *p*-phenylenediamine, rhabdomyolysis

## Abstract

Rhabdomyolysis is a disorder characterized by acute damage of the sarcolemma of the skeletal muscle leading to release of potentially toxic muscle cell components into the circulation, most notably creatine phosphokinase (CK) and myoglobulin, and is frequently accompanied by myoglobinuria. In the present work, we evaluated the toxicity of *p*-phenylenediamine (PPD), a main component of hair dyes which is reported to induce rhabdomyolysis. We studied the metabolic effect of this compound *in vivo* with Wistar rats and *in vitro* with C2C12 muscle cells. To this aim we have combined multi-omic experimental measurements with computational approaches using model-driven methods. The integrative study presented here has unveiled the metabolic disorders associated to PPD exposure that may underlay the aberrant metabolism observed in rhabdomyolys disease. Animals treated with lower doses of PPD (10 and 20 mg/kg) showed depressed activity and myoglobinuria after 10 h of treatment. We measured the serum levels of aspartate aminotransferase (AST), alanine aminotransferase (ALT), and creatine kinase (CK) in rats after 24, 48, and 72 h of PPD exposure. At all times, treatment with PPD at higher doses (40 and 60 mg/kg) showed an increase of AST and ALT, and also an increase of lactate dehydrogenase (LDH) and CK after 24 h. Blood packed cell volume and hemoglobin levels, as well as organs weight at 48 and 72 h, were also measured. No significant differences were observed in these parameters under any condition. PPD induce cell cycle arrest in S phase and apoptosis (40% or early apoptotic cells) on *mus musculus* mouse C2C12 cells after 24 h of treatment. Incubation of *mus musculus* mouse C2C12 cells with [1,2-^13^C_2_]-glucose during 24 h, subsequent quantification of ^13^C isotopologues distribution in key metabolites of glucose metabolic network and a computational fluxomic analysis using in-house developed software (Isodyn) showed that PPD is inhibiting glycolysis, non-oxidative pentose phosphate pathway, glycogen turnover, and ATPAse reaction leading to a reduction in ATP synthesis. These findings unveil the glucose metabolism collapse, which is consistent with a decrease in cell viability observed in PPD-treated C2C12 cells and with the myoglubinuria and other effects observed in Wistar Rats treated with PPD. These findings shed new light on muscle dysfunction associated to PPD exposure, opening new avenues for cost-effective therapies in Rhabdomyolysis disease.

## Introduction

Rhabdomyolysis is a degenerative muscle disease, whose primary symptoms are muscle weakness, cramps, sweating, and nausea, mainly because of muscle cells apoptosis (Melli et al., 2005; Wilson, 2006). Although the multi-factorial nature of rhabdomyolysis, a number of studies indicate that cell injury may be mediated by a final common pathway (Melli et al., 2005). Thus, a higher cellular permeability to sodium ions associated to rhabdomyolysis can be triggered by diverse noxious factor, such as toxins or drugs activating cytolytic enzymes, injuring the cellular membrane, dysregulating metabolism and disrupting the cell integrity, hypoxic conditions reducing energy production in the cell, among others. Cytoplasmic sodium accumulation increases cytosolic and mitochondrial calcium levels which activates proteolytic enzymes affecting cell membrane integrity and ultimately releasing cellular components to the circulation. Thus, some of these components, such as myoglobin or creatine kinase, facilitate clinical recognition of rhabdomyolysis (Melli et al., 2005).

A variety of studies report the capability of *p*-phenylenediamine (PPD) to induce skeletal muscle lesions in rats. PPD is one of the main components of hair dyes, which have been widely used to investigate the inflammatory and degenerative pattern of muscle fibers (Garrigue et al., 2006; Pourahmad et al., 2008; Zoll et al., 2015). PPD, together with several of its amino alkyl derivates are known to be myotoxic in animals and humans, causing necrosis of cardiac, and/or skeletal muscle (Iannotti et al., 2010). To clarify the mechanism of PPD related occurrence of rhabdomyolisys, we investigated the pharmacological effects of PPD on the contractile proteins and the sarcoplasmic reticulum (SR) in single skeletal mice muscle fibers by using the skinned fiber method (Giulivi et al., 2011). Thus, it was speculated that PPD can bring out rhabdomyolisys by promoting the calcium-induced calcium release (CICR) and leakage of Ca^2+^ from the SR, increasing Ca^2+^ concentration and triggering subsequent changes in the muscle, such as irreversible alterations in muscle structure, continuous contraction, and/or metabolic changes (Giulivi et al., 2011).

PPD toxicity, and more specifically its role in rhabdomyolysis initiation, has been demonstrated by experiments conducted by administering PPD to mice (Chwaluk, 2013) that in turns alters creatine kinase (CK) levels in blood (Wilson, 2006).

In the present work, we assessed the toxicity of PPD, the antiproliferative effect on C2C12 cells by using Annexin V-Fluorescein Isothiocyanate binding assay, the capacity to induce cell cycle arrest, and apoptosis by applying FACS (fluorescence-activated cell sorter) and the molecular mechanisms triggered by this compound inducing rhabdiomyolysis in Wistar Rats and in C2C12 cells by applying tracer-based metabolomic approaches.

Metabolism, as an effector of multiple cellular signals, represents the end point of many cellular events. Thus, metabolic characterization is a powerful tool to enhance our understanding of how compounds such as PPD exert their function on muscular cells *in vitro*. In this sense, the use of tracer-based metabolic substrates (i.e., [1,2-^13^C_2_]-glucose) combined with mass spectrometry (MS) permits to infer the flux through the main metabolic reactions involved in the biosynthetic metabolism and energy production of the cell. This approach has been widely used in a number of studies determine the metabolic flux profile of a variety of *in vitro* models (Boren et al., 2003; Vizan et al., 2005; Burchert, 2007; Kominsky et al., 2009). More specifically, metabolic isotope distribution analysis (MIDA) or metabolic flux analysis via model-driven approaches permit the simulation and evaluation of substrate flux through major metabolic pathways under various physiological conditions (Cowan-Jacob et al., 2004). In order to correlate rhabdomyolysis and apoptosis associated to PPD with characteristic metabolic patterns, we modeled glucose metabolic network using available enzyme kinetic data, the experimentally measured ^13^C isotopomer distribution data and the previously developed software Isodyn (Selivanov et al., 2004, 2005, 2006). This approach enabled evaluating the metabolic flux profiles in the analyzed metabolic network, which were supported by the mass isotopomers distribution obtained after incubation with labeled glucose in the presence of the PPD compound.

## Materials and methods

### *In vivo* assays with wistar rats; test animals and housing

The study was approved by the Ethics Committee on Animal Experimentation of University of Navarra. Wistar female rats, with a body weight of 160 g ± 20% (8 weeks old), were supplied by Harlan (Harlan Laboratories, Indianapolis, IN). The animals were housed in polycarbonate cages with stainless steel covers in a room under controlled temperature conditions (22 ± 3°C), controlled relative humidity (50 ± 20%) and light (12 h light–dark cycle). Air was exchanged 15 times/h sterile food supplied by Harlan (Harlan Laboratories, Indianapolis, IN) and controlled water was available *ad libitum*. The rats were housed in the study room for 5 days prior to the beginning of treatment in order to allow acclimatization to the environmental conditions. Finally, three rats were randomly assigned to each studied group corresponding to different PPD doses (10, 20, 40, and 60 mg/Kg) and control (DMSO).

### Dosage and observations

**Figure 1:**
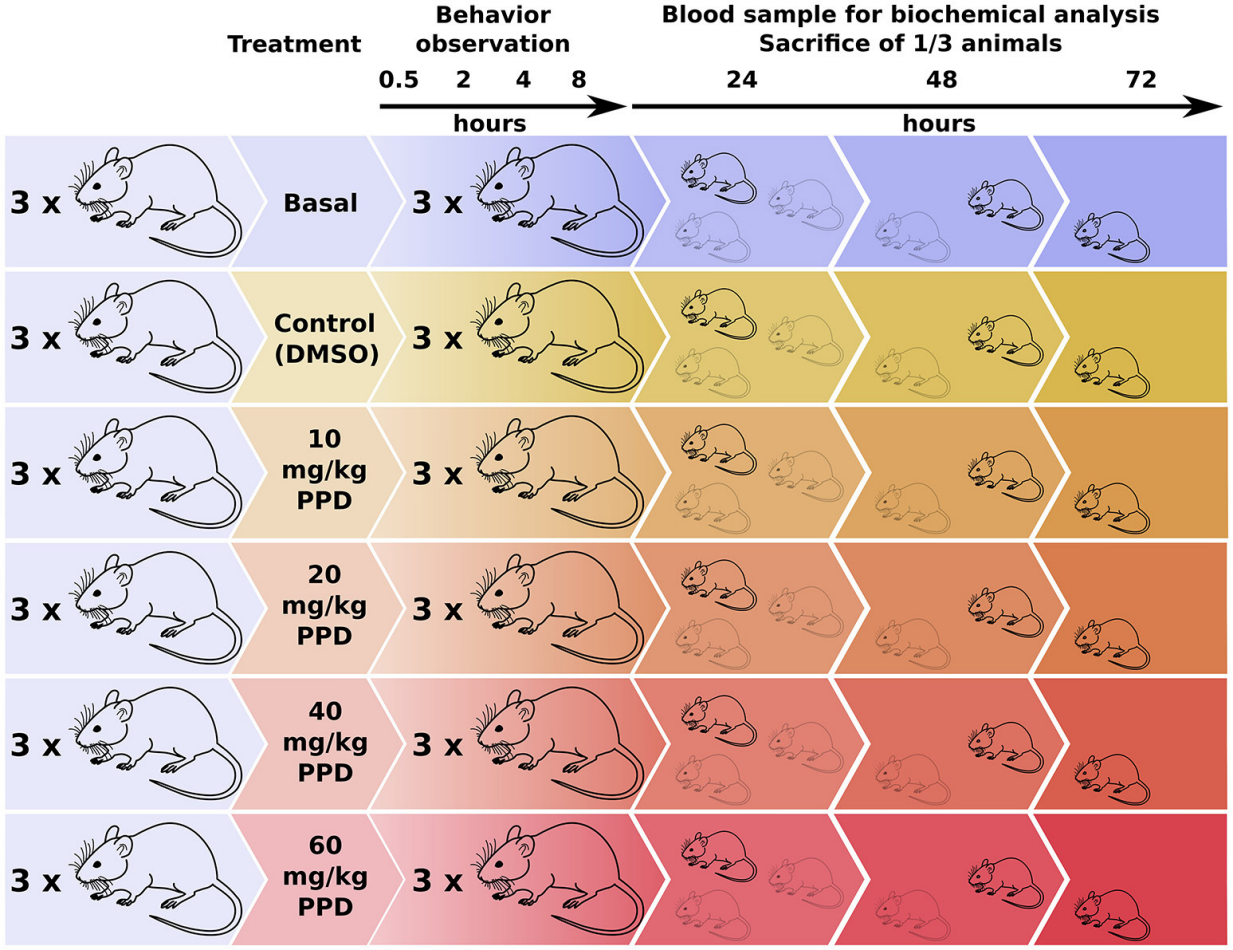
**Experimental design for the dose-effect *in vivo* studies of PPD on Wistar Rats**. Rats were divided in one basal group, one control group (only DMSO administered) and four groups with an increasing PPD concentration with three individuals per group. Rats' behavior is observed at different time points until 8 h after treatment. Next, blood sample is extracted from one mice of each group and it is sacrificed for tissue and organ examination. This process is repeated at 24, 48, and 72 h after treatment.

The animals were divided into groups of three rats at random. A control group receiving the vehicle and four groups receiving PPD at several concentrations 10, 20, 40, and 60 mg/kg at single dose were included (Figure 1). The compound was administered in a volume of 300 μl of 100% DMSO by gavage. Animals were evaluated based on general appearance, behavior and signs of morbidity using the Irwin test (Castagné et al., 2012), immediately following administration, 30 min, 1, 2, 4, and 8 h after the administration (Figure 1). All of the animals were weighed just before the acclimatization period, before administration and 24, 48, and 72 h after administration. At the end of the observation period (24, 48, and 72 h after administration), all the animals were anesthetized and blood samples were collected from tail and retro orbital sinus and centrifuged at 2000 × g for 30 min at 4°C to recover the serum (Figure 1). The serum was frozen and stored at −20°C until determination of biochemical parameters such as urea, creatinin, Glucose, aspartate aminotransferase (AST), alanine aminotransferase (ALT) and creatine kinase (CK), lactate dehydrogenase (LDH), at 24, 48, and 72 h before sacrificed in a CO_2_ chamber and autopsied. All of the tissues were observed grossly. Ovaries, kidneys, liver, spleen, heart, and lung were weighed and the relative organ weights were calculated. Serum CK and LDH levels were measured.

### Cell culture

*C2C12* cells from *Mus musculus* mouse were cultured in DMEM (Dulbecco's Modified Eagle's medium) with 10 mg/ml of streptomycin, 2 mM glutamine, 10,000 units/ml of penicillin and 10% heat-inactivated FCS, until subconfluence was reached. For biochemical characterization, C2C12 cells were cultured in DMEM, supplemented with 4 mM asparagine without glutamine, 10 mg/ml of streptomycin, 10,000 units/ml of penicillin and 10% heat-inactivated FCS.

### Cell viability assay

The absorbance of MTT [3-(4,5-Dimethylthiazol-2-Yl)-2,5-Diphenyltetrazolium Bromide] dye staining of living cells was used to determine cell viability (Pachon et al., 2007; Pachón et al., 2008). Here, 3.5 × 103 C2C12 cells/well were cultured in 96 well-plates at different PPD concentrations (from 1 to 100 μg/ml). An ELISA plate reader (Tecan Sunrise MR20-301, TECAN, Austria) was used to determine the relative cell viability, the absorbance was determined at 550 nm. Cell survival rate relative to untreated cells after 24 h of treatment was used to estimate the concentrations that caused 50% (IC_50_) and 80% (IC_80_) of inhibition of C2C12 at that time.

### Cell cycle analysis

Cell cycle was evaluated by measuring FACS (fluorescence-activated cell sorter) in an Epics XL flow cytometer at 488 nm (Coulter Corporation, Hialeah, FL, USA). This technique allows for the semiquantitation of DNA in each cell and permits to separate cells in G0 or G1 phase (one copy of DNA), G2 or M phase (after DNA duplication) and S phase (the amount of DNA per cell is in an intermediate state between the two previous conditions). For these measurements, C2C12 cells were cultivated in 6 well-plates (4 × 10^5^ cells/well each) with 2 ml of culture medium. After 24 h of plated, PPD corresponding to IC_50_ and IC_80_ concentrations was added and cells were incubated for 24 h, in parallel, control C2C12 cells were cultured without PPD. All experiments were performed five times with two replicates for each experiment.

### Annexin V-Fluorescein isothiocyanate binding assay

This technique permits to determine whether a cell is at early or late apoptosis or necrotic state. In early apoptosis, Phosphatidylserine (PS) is translocated to the outer membrane allowing PS-annexin V-Fluorescein Isothiocyanate (FITC) binding. In late apoptosis, as well as in necrotic processes, cell membranes lose their integrity allowing PI to access the nucleus and bind the DNA bases. Next, by using FACS analysis one can determine PI and annexin V-FITC staining accumulation to differentiate non-apoptotic cells (PI– and annexin V–) from early apoptotic (PI− and annexin V+) and necrotic or late apoptotic (PI+) cells (Cortés et al., 2012). The same amount of C2C12 cells mentioned for cell cycle assay was treated with PPD as previously described. Cells were then collected and resuspended in binding buffer (10 mM Hepes/NaOH, pH7.4, 140 mM NaCl, 2.5 mM CaCl_2_). Annexin V-FITC conjugate (1 μg/ml) was added and incubated for 30 min at room temperature in the dark. Just before FACS analysis [Bender medSystems (Innogenetics) human Annexin V-FITC Kit cat No. BMS306FI], cells were stained with 1 mg/ml PI solution. In each experiment ~20 × 10^3^ cells per condition were analyzed and the experiment was performed five times.

### Statistics

Data is represented as the mean value from independent experiments ± SEM. The statistical analysis, comparing the threated and the control untreated cells, was carried out by using the Student's *t*-test. This is a widely used statistical analysis to compare two populations' means with unknown variance and assuming normal distribution.

### Biochemical characterization of C2C12 cells

Glucose and lactate concentrations were determined from culture medium at initial and end-time points by using Cobas Mira Plus chemistry analyzer (HORIBA ABX, Montpellier, France; Cisternas et al., 2016).

### Metabolic profile of C2C12

To track and measure the substrate carbon flux through central carbon metabolic pathways of C2C12 cells when treated with PPD, mass isotopomer distribution analysis (MIDA), and metabolic modeling approaches were used.

#### Sample preparation

Cells were cultured in DMEM (Biowithaker) °supplemented with 10% dialyzed heat-inactivated Fetal Bovine Serum (FBS), glucose (10 mM, 50% isotope enrichment [1,2-^13^C_2_]-glucose), asparagine (20 mg/ml), penicillin (100 U/ml), and streptomycin (100 μg/ml). PPD was added to the flasks at the IC_50_ concentration determined for 24 h (154 μM), in parallel, control C2C12 cells were cultured without PPD. Initially each flask contained 10^6^ cells and the incubation was carried out for 24 h. To separate medium and cell pellets, cells were centrifuged at the end of incubation (3000 × g, 5 min). Afterwards, pellet and medium were stored at −20°C for metabolite isolations.

#### Metabolite isolation and derivatization

The following metabolites were isolated and derivatized for their quantification in PPD-treated and non-treated C2C12 cells:

Glucose. A mixed DOWEX 50-DOWEX 1 ionic exchange column was used to corroborate that the original medium contained 50% of labeled glucose ([1,2-^13^C_2_] D-glucose). First, 500 μl of medium and after 6 ml of milliQ water were passed through the column and were collected into a glass tube. Next, it was lyophilized for 12 h to eliminate the remaining liquid and the dried sample was stored. Finally, hydroxyl-amine in pyridine and acetic anhydride were used to derivatize glucose to its aldonitrile form. The ion cluster was set to m/z 328 (C1–C6, chemical ionization; Wamelink et al., 2008).
Glycogen. Glycogen for biochemical and isotopomerics distribution determination was extracted as described in Burchert (2007), by direct digestion of sonicated extracts with amyloglucosidase. Measurement of glucose released from glycogen was done by using the isotopomer [U-13C6-1,2,3,4,5,6,6-D7]D-glucose (or [U-13C-D7]-glucose as shorter name) as recovery standard and internal standard quantification procedures. The hydrolyzed glucose was isolated using ion exchange chromatography (Mao et al., 2002) and was derivatized to its aldonitrile acetate form using hydroxyl-amine in pyridine and acetic anhydride. We monitored the ion cluster around the m/z 328 (C1–C6, chemical ionization; Wamelink et al., 2008).
Lactate. Lactate from the culture media of C2C12 cells, which had been cultured and treated as explained above, was extracted by ethyl acetate. In more detail, 1 ml of culture medium was acidified with 2–3 drops of HCl, followed by addition of 1 ml of ethyl acetate and a little amount of sodium chloride and 1 min shaken. Next, the samples rest for 2 min and were centrifugated at 4,000 rpm for 10 min in a Jouan B3.11 centrifuge. Next, organic phase was transferred to a tube and the remaining ethyl acetate was removed by applying aa slight N2 pressure for 10–15. Lactate was derivatized to its propylamideheptafluorobutyric form. Next, the m/z 328 was monitored (C1–C3, chemical ionization, Wamelink et al., 2008).
Ribose from RNA. Acid hydrolysis of the cellular RNA was used to isolate ribose from C2C12 treated and non-treated cells with the fractions as indicated above. First, 1 ml of Trizol was added and dissolved by sonication (30 s) to the cold pellets, next, it was transferred to free RNase eppendorfs and 200 μl of chloroform were added. Next, the samples were shaken and centrifuged in an Eppendorf microfuge for 30 min at 14,000 rpm at 4°C. Chloroform and RNA from the non-colored upper phase was collected and 1 ml of cold 2-propanol was added and the mixture was reposing overnight to precipitate the DNA. Next, samples were centrifuged as indicated above. RNA was further purified by removing the supernatant, next 1 ml of 75% cold ethanol was added. The samples were shaken and centrifuged to eliminate the supernatant and re-suspended with 10 μl of miliQ water and 2 ml of HCl 2N. Next, the samples were heated at 100°C for 2 h, cooled at room temperature (acid hydrolysis of RNA) and dried with air preasured to eliminate the remaining liquid or stored stored at −80°C. Acetic anhydride and hydroxyl-amine in pyridine were used to derivatize ribose to the aldonitrile acetate form. Finally, the 13C molar enrichment in ribose was quantified by setting the ion cluster at m/z 256 (C1–C5, chemical ionization, Wamelink et al., 2008).
Glutamate. Glutamate was extracted from the culture media of cultured and treated C2C12 cells by using ammoniac and acetic acid. In more detail, 1 ml of culture medium was passed through the previously prepared ionic exchange column DOWEX 50. Next, 6 ml of milliQ water and after 5 ml of NH_4_OH 2N were passed through the column, the volume was collected into a glass tube and immediately dried eliminating the remaining liquid by air pressure. Then, 6 ml of milliQ water were added to the tube and passed through to DOWEX 1 ionic exchange column earlier prepared, Next, 5 ml of acetic acid CH_3_COOH 0.5N were also added and the total volume was collected. Finally, samples were dried with air pressure for 12 h and stored for determination. Glutamate was derivatized to its nitrifluoroacetyl-n-butyl form The ion clusters m/z 198 and 152 (C2–C5 and C2–C4 electron impact ionization, respectively) were monitored. Isotopomeric analysis of glutamate C2–C5 and C2–C4 fragments from cultured medium to infer the relative contributions of pyruvate dehydrogenase and pyruvate carboxylase to the tricarboxylic acid (TCA) cycle (Cowan-Jacob et al., 2004; Yoo et al., 2008).
Gas Chomatography/Mass Spectrometry (GC/MS). Mass spectral data were obtained on the HP5973 mass selective detector connected to an HP6890 gas chromatography. The settings were as follows: GC inlet 230°C, transfer line 280°C, MS source 230°C, MS Quad 150°C. An HP-5 capillary column (30 m length, 250 μm diameter, 0.25 μm film thickness) was used for glucose, lactate, ribose, and glutamate analysis (Wamelink et al., 2008). A Bpx70 column (25-m length, 220-μm diameter, 0.25-film thickness; SGE Incorporated, Austin, TX) was used for fatty acids analysis with specific temperature programming for each compound studied.

### Computational modeling of tracer-based metabolic data

**Figure 2:**
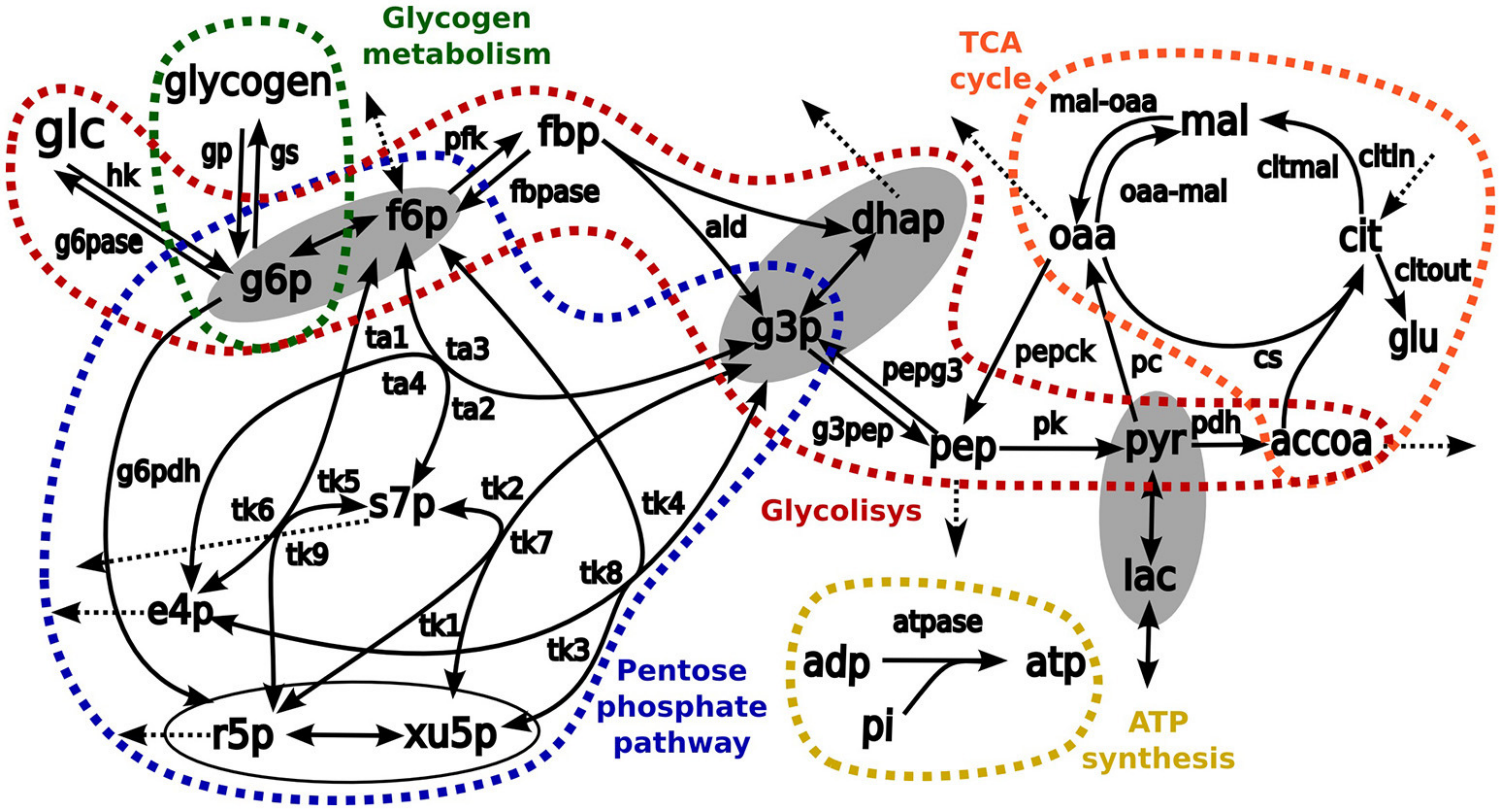
**The schemes of kinetic model used as a base for simulation of isotopologue distribution**. Arrows represent biochemical reactions connecting metabolites. Pathways considered in the model are enclosed and highlighted in different colors: red, glycolysis; blue, pentose phosphate pathways; orange, TCA cycle, and green glycogen metabolism. The metabolites enclosed in ellipses are considered to be in fast equilibrium. List of abbreviations for metabolites: glu, glutamate; glc, glucose; glgn, glycogen; lac, lactate; g6p, glucose 6 phosphate; fbp, fructose 1,6-bisphosphate; f6p, fructose 6-phosphate; g3p, glyceraldehyde 3-phosphate; dhap, dihydroxyacetone phosphate; pyr, pyruvate; accoa, acetyl coenzyme A; pep, phosphoenolpyruvate; e4p, erythrose 4-phosphate; r5p, ribose 5-phosphate; s7p, sedoheptulose 7-phosphate; xu5p, xylulose 5-phosphate; cit, citrate; oaa, oxaloacetic acid; mal, malate; adp, adenosine diphosphate, and atp, adenosine triphosphate. Enzymes: hk, hexokinase; g6pase, glucose 6-phosphatase; g6pdh: glucose 6-phosphate dehydrogenase; gp, glycogen phosphorylase; gs, glycogen synthase; pfk, phosphofructokinase; fbp, fructose 1,6-bisphosphatase; ald, aldolase; pk, pyruvate kinase; pepck, phosphoenolpyruvate carboxykinase; pdh, pyruvate dehydrogenase complex; pc, pyruvate carboxylase; cs, citrate synthase; atpase, ATP synthesis; pepg3, pathway pep→g3p; g3pep, pathway g3p→pep; mal-oaa, pathway mal→oaa; oaa-mal, pathway oaa→mal; transaldolase activity, ta1, f6p→s7p; ta2: s7p→f6p; ta3, f6p↔g3p; and ta4, s7p↔e4p; transketolase activity: tk1, x5p→s7p; tk2, s7p→x5p; tk3, f6p→x5p; tk4, x5p→f6p; tk5, f6p→s7p; tk6, s7p→f6p; tk7, x5p↔g3p; tk8, f6p↔e4p; and tk9, r5p↔s7p.

Transfer of ^13^C carbons from medium [1,2-^13^C2]-glucose into intracellular metabolites was simulated by using in-house developed software for stable isotope tracer data analysis (Selivanov et al., 2004, 2005, 2006; Isodyn, Marin de Mas et al., 2011). The simulation scheme included all isotope-exchange reactions in pentose phosphate pathway, TCA cycle, glycolysis, anaplerotic reactions, exchange with extracellular lactate, glutamate and glucose, ATP metabolism, and biosynthetic fluxes (Figure 2). In brief, the software iteratively finds a set of parameters that defines a metabolic flux profile to simulate the metabolic label distribution that best fits the experimental measurements. Finally, to evaluate the reliability of the analysis, χ^2^ statistic is used to compare computed and measured label distributions as is described in Equation (1). Here, *y*_*i*_ is the experimental fraction measurement of the *i*th isotope of metabolite *y, y(x*_*i*_*,a*) is the computed fraction of the same isotope and σ_*i*_ is the standard deviation of the *i*th isotope of metabolite y.

(1) $$\chi^{2}={\sum_{i\;=\;1}^{N}\left[\frac{y_{i}-y\text{​}\left(x_{i},a\right)}{\sigma_{i}}\right]}^{2}$$

This software enables to integrate tracer-based metabolic data and metabolite consumption/production rates to infer the internal metabolic fluxes in control and PPD-treated cells. A more detailed description of Isodyn software is in the Supplementary Material 2.

### Data analysis and statistical methods

Three cultures for each treatment were used for the *in vitro* experiments. In mass spectral analyses, three independent automatic injections were performed for each sample. Sample measurements with a standard deviation higher than 1% of the normalized peak intensity were rejected. Level of significance was determining by performing Mann-Whitney *U*-test, with two independent samples (three replicates of each one of the two samples, having six measurements in total). This is a non-parametric test applied to compare two independent sample populations. Differences in glucose carbon metabolism between control and PPD with an associated *p* ≤ 0.05 were considered as significant.

## Results

### PPD presents toxic effects and alters the molecular biomarkers in blood on wistar rats

Increasing doses of PPD (from 10 mg/Kg of body weight to 60 mg/kg of body weight) was administered to Wistar female rats. Immediately after administration, all of the animals showed a decreased spontaneous reactivity, which lasted between 10 h and 2 days, depending on the doses (Table 1). These symptoms were not observed in the control animals. The evolution of body weight was normal in all groups. Changes in the general state, external appearance or behavior were observed along the observation period in animals treated with PPD and a general depressed activity and piloerection which lasted from 10 h until death, depending on the doses (Table 1). Animals treated with PPD at lower doses (10 and 20 mg/kg) until 10 h of treatment, showed depressed activity and some of they had brown color urine or myoglobinuria. Animals treated with higher doses of PPD (40 and 60 mg/kg) until 4 h of treatment, showed total loss of equilibrium, hypotonic gait and piloerection. After 48 h of treatment with PPD at 60 mg/kg, mices they ataxic gait, but this effect disappeared after 72 h of treatment. After 48 and 72 h of treatment, one animal of each group of PPD treatment was sacrificed and necropsied.

**Table 1:** **Toxicity after oral administration of four concentrations of PPD compound in mg/kg**.

**Compound**	**Doses**	**Hipoactivity**	**Piloerection**	**Ataxic. G**	**Hypot. G**	**Myoglo**
PPD	10	10 h	2–4 h	0/3	0/3	++
	20	10 h	2–4 h	0/3	0/3	++
	40	1 day	Until death	2/3	0/3	
	60	2 days	Until death	0/3	3/3	
DMSO	300 μl	30 min	1 h	0/3	0/3	

*The compound was dissolved in DMSO and 300 μl of solution was administered. Animals were sacrificed after 48 and 72 h of observational period. Periods of time are in hours (h), days or minutes (min). ++ represents the presence of myoglobinuria; Ataxic G, Ataxic Gait; Hypot G, Hypotonic Gait; Myoglo, Myoglobiunuria*.

**Figure 3:**
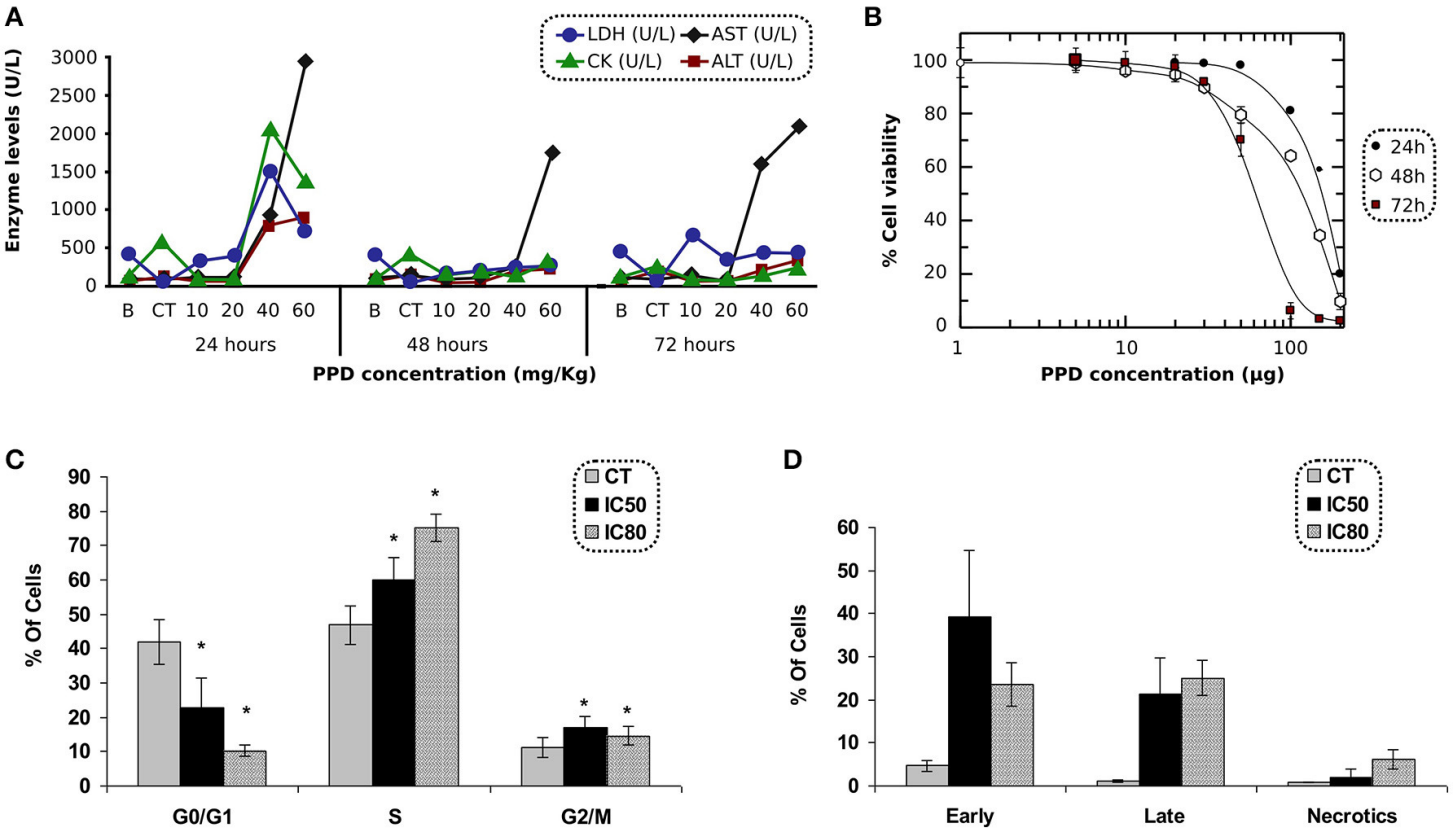
**(A)** LDH, CK, AST, and ALT levels after 24, 48, and 72 h of PPD treatment at different doses administration in Wistar rats, control group (CT) and the enzyme levels before PPD administration (B). The measurements are given in U/L (International unit of enzymatic activity per liter). **(B)** Effect of PPD doses (in μg) on cell proliferation of C2C12 cells, after 24, 48, and 72 h of incubation. The relative percentage of cell proliferation was calculated assuming a 100% of proliferation in untreated cells at 24, 48, and 72 h. Each point represents the mean of triplicate experiments. **(C)** Cell cycle analysis of C2C12 treated with PPD. C2C12 cells were exposed to PPD for 24 h at 37°C with either IC_50_ or IC_80_ (second and third column, respectively) concentrations, or the solvent (first column as control) were stained with propidium iodide to conduct the cell cycle analysis of five independent experiments. Values are represented as the mean ± *SE*. ^*^Significantly different at *p* < 0.05 compared with control cells. **(D)** C2C12 cells were stained with propidium iodide accumulation and Annexin V-FITC and exposed to PPD in the flow cytometry analysis. The concentrations used in this study corresponded to IC_50_ and IC_80_ (second and third column, respectively), or the solvent (first column as control) for 24 h. Results are mean ± *SD* of five independents experiments.

Biochemical parameters in blood were measured at 24, 48, and 72 h after PPD dosage. Results obtained with animals treated with PPD compound indicate that AST and ALT (Figure 3A) increased at 24, 48, and 72 h, and LDH and CK enzymes (Figure 3A) increased at 24 h after the final dose of treatment at higher doses of PPD (40 and 60 mg/kg).

Blood packed cell volume and hemoglobin levels, as well as organs weight at 48 and 72 h, were also measured. No significant differences were observed in these parameters under any condition. Histological analysis of the different extracted organs showed patterns similar to controls.

### Antiproliferative effect of PPD on C2C12 cells

The antiproliferative effect of PPD was assessed in C2C12 cells after 24 h of incubation. The IC_50_ concentrations were obtained by MTT test. PPD concentrations were plotted against the percentage of cell proliferation after 24, 48, and 72 h of incubation. The IC_50_-values obtained in C2C12 cells were 154 ± 6, 108 ± 8, and 59 ± 1 μM at 24, 48, and 72 h, respectively and the IC_80_-values were 201 ± 8, 182 ± 12, and 91 ± 7 μM at 24, 48, and 72 h, respectively (Figure 3B).

#### Cell cycle and apoptosis

C2C12 cells treated with PPD at IC_50_ and IC_80_ concentrations after 24 h of treatment showed an increase in the population in S phase (13% at IC_50_, and 28% at IC_80_ for C2C12 cells, respectively) compared to untreated cells. It was accompanied by a concomitant decrease in the percentage of cells in the G0/G1 phases (19% at IC_50_, and 32% at IC_80_ for C2C12 cells), suggesting an arrest at the S-phase in PPD-treated cells (Figure 3C).

Apoptosis in C2C12 cells was assessed after 24 h of treatment with PPD using the concentrations mentioned in cell cycle analysis. We differentiate late apoptotic/necrotic cells (annexin PI^−^ and V^+^) from early apoptotic cells (annexin V^+^ and PI^−^) by performing FACS analysis using annexin V-FITC staining and PI accumulation. Results showed that C2C12 cells treated with PPD at IC_50_ concentration increase apoptosis in 55% (34.7 and 18.8% of early and late apoptosis, respectively) with respect to the untreated cells. We also observed that cells treated with PPD at IC_80_ concentration increased apoptosis in 43% (20.1% early apoptosis plus 23.9% late apoptosis) compared with non-treated C2C12 cells (Figure 3D). Annexin-V binding assay showed that PPD induced a high number of apoptotic cells.

### Determining differences in central carbon metabolism activity in C2C12 cells treated with PPD via mass isotopomer distribution analysis (MIDA)

#### Glucose

**Figure 4:**
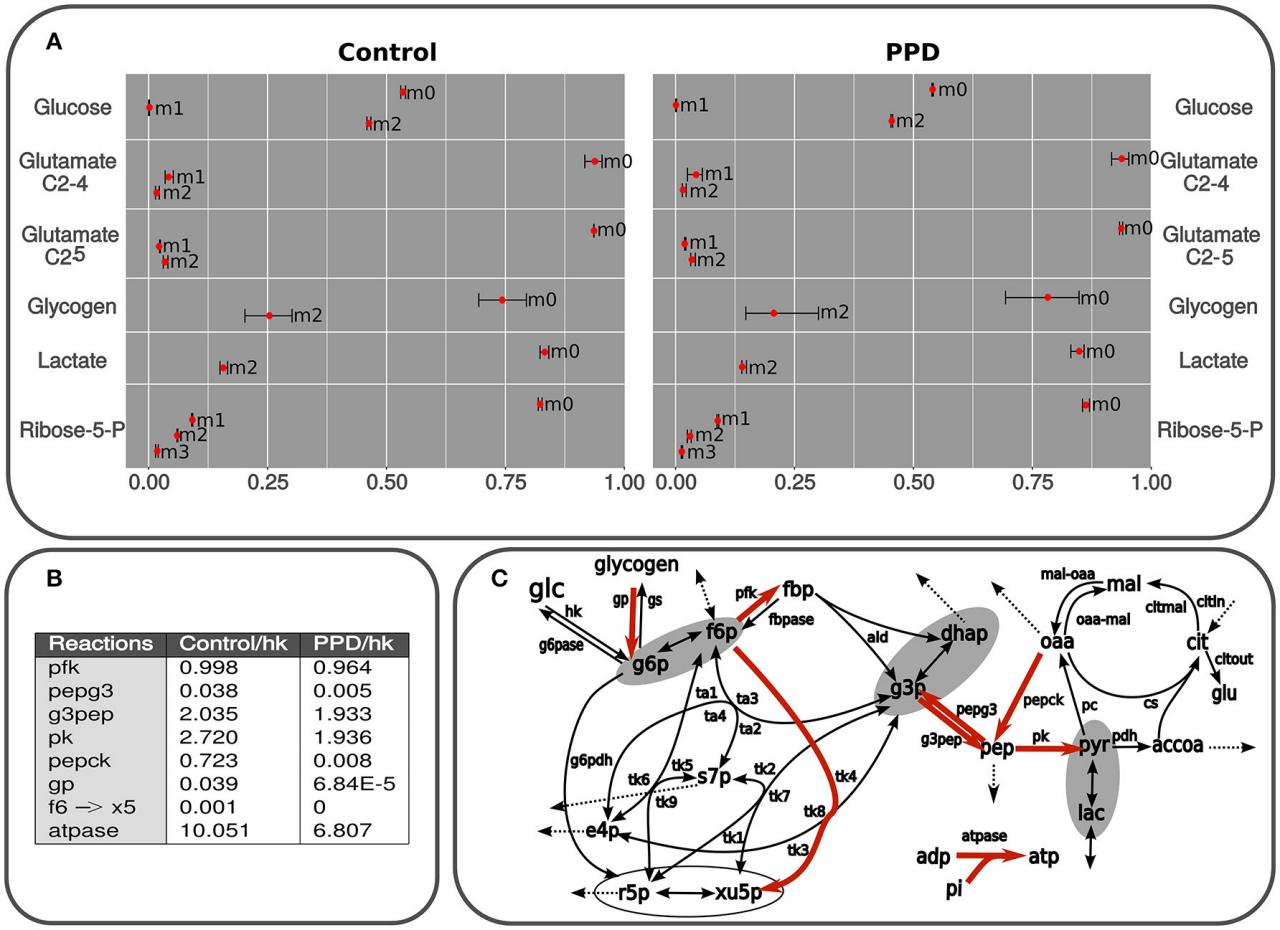
**(A)** Isotopologues are described as follows: non-labeled (m0), m1, containing one ^13^C isotope (m1), m2 (two ^13^C isotopes (m2), etc. Predicted and measured isotopologues from isolated C2C12 cells using glucose as the only substrate and containing 50% of [1,2-^13^C2]D-glucose. The results correspond to lactate, glutamate, and glucose from medium, glucose from glycogen after 24 h exposed to PPD and non-exposed (control) cells. The measurements are presented as mean ± standard deviation and the predicted label distribution for each isotopologue is represented with red points. The data was simulated using a model in accordance with the scheme presented in Figure 2. The fitting was carried out by applying an algorithm described in Marin de Mas et al. (2011). The difference between experimental data and the best fit (Section Materials and Methods) are summarized for the whole set of data. The whole isotope distribution can be found in the Supplementary Material 1. **(B)** Principal fluxes of central carbon metabolism estimated in control and PPD-treated C2C12 cells. This table shows the fluxes with significant differences between groups (*p* < 0.05). Fluxes are expressed as mmol × mL^−1^ min^−1^ × flux hk. Abbreviations used in this table correspond to flux notation in Figure 2. The whole isotope distribution can be found in the Supplementary Material 1. **(C)** Graphical representation of the kinetic model used for isotopologue distribution simulation. Principal fluxes of central carbon metabolism estimated in control and PPD-treated C2C12 cells. Metabolic reactions highlighted in red represent predicted fluxes significantly higher in control cells (no reactions with higher flux in PPD-treated group were predicted). Abbreviations used in this figure correspond to flux notation in Figure 1.

Glucose consumption and lactate production were determined by using spectrophotometric methods following 24 h of incubation of C2C12 cells treated with PPD at IC_50_. The results didn't show differences between the initial and the final ^13^C enrichment in glucose (46.3% of [1,2-^13^C_2_]-glucose, glucose m1 in Figure 4A -m0, non-labeled; m1, containing one ^13^C isotope; m2, two ^13^C isotopes, etc…), which indicates that no glucose was released.

#### Lactate

An approximate estimation of the metabolic flux through the pentose phosphate pathway (ppp) and glycolysis can be inferred by measuring the ^13^C label distribution in lactate (Lactate isotopologue distribution is depicted in Figure 4A). The two ^13^C labeled lactate (m2 in Figure 4A) is directly produced via glycolysis, whereas one ^13^C labeled lactate (m1 in Figure 4A) is the product of the oxidation of [1,2-^13^C_2_]-glucose via oxidative branch of pentose-phosphate pathway and then recycled to glycolysis through the non-oxidative branch of pentose-phosphate pathway (Wamelink et al., 2008). Thus, the percentage of glucose metabolized directly to lactate can be determined by applying the formula: Glycolitic rate = [Δlactate × m2_lactate_]/[Δglucose × m2_glucose_], where Δglucose is the glucose consumption (Control: 2.31 ± 0.04 mM; PPD: 2.42 ± 0.26 mM) and Δlactate is the lactate production (Control: 2.5 ± 1.0 mM; PPD: 1.5 ± 0.3 mM). Here, we didn't find significant differences in the glycolitic rate between the PPD-treated and control cells (28.8 ± 14 and 31.9 ± 13%, respectively). Additionally, we did not observe significant enrichment in m1 lactate isotopomer which indicates a low activity of ppp and that glucose was metabolized to lactate mainly through glycolysis in both groups. The results also showed that glycolytic contribution to lactate production is higher in non-treated C2C12 cells.

#### Glycogen

We didn't observe significant differences in the total glycogen reservoirs in the C2C12 cells after 24 h of incubation in none of the groups (10.1 μg of glycogen). Furthermore, significant ^13^C incorporation was observed in glycogen after incubation. More specifically glycogen was enriched in m2 species in both groups (glycogen m2 in Figure 4A), which suggests an important exchange between medium [1,2-^13^C_2_]-glucose, and glycogen reservoirs. On the other hand, m1 isotopologue was no significant in both groups (glycogen m1 in Figure 4A) which suggest a low contribution of ppp to glycogen metabolism in C2C12 cells and is supported by our previous analysis of glucose and lactate labeling data.

#### Ribose

To estimate the nucleic acid precursor synthesis in C2C12 cells treated with PPD, measurements of the molar enrichment of RNA ribose from [1,2-^13^C_2_]-glucose were carried out by calculating the overall ^13^C enrichment by applying the following formula: Σm_n_ = m1 + 2 × m2 + 3 × m3 + 4 × m4 + 5 × m5. This calculation showed that Σm_n_ ribose label enrichment significantly decrease after treatment with PPD (0.294 ± 0.028 in control and 0.197 ± 0.023 in PPD-treated cells), which indicates an inhibition of *de novo* nucleic acid synthesis in treated cells compared with control cells.

Additionally, we found significant differences in the balance between non-oxidative and oxidative branch of pentose-phosphate pathway between control and PPD-treated cells. It was determined by calculating the ratio between both branches of ppp as is described in Wamelink et al. (2008): ox/non-ox = m1 + m3 / (m2 + m3 + 2m4). Here, we found that this ratio was 1.183 ± 0.004 in control cells and 2.224 ± 0.189 in PPD-treated cells. These differences are mainly due to the low activity of the non-oxidative branch of pentose-phosphate pathway in PPD-treated cells as the glucose consumption is driven to lactate production and no r5p is recycled to glycolysis. Besides, the activity of the oxidative branch of pentose-phosphate pathway is also lower in PPD treated cell decreasing the molar enrichment in ribose.

Moreover, these results correlate with the fact that m1 lactate isotopomer was almost undetected (Supplementary Material 1) in both groups, suggesting that in both conditions glycolysis is the preferred pathway to glucose consumption.

#### Glutamate

Glutamate, a non-essential amino-acid, is partially produced from mitochondrial α-ketoglutarate, which is a central intermediate of the TCA cycle. Therefore, label incorporation from glucose into glutamate is a good indicator of TCA cycle anabolic metabolism for amino acid synthesis instead of glucose oxidation (Wamelink et al., 2008). The analysis did not reveal significant ^13^C enrichment in glutamate in any of both C2C12 cells groups.

### Unveiling metabolic flux alterations in C2C12 cells treated with PPD via model-driven method

In order to perform a more in-depth analysis of the tracer-based metabolic data and to identify the key metabolic adaptations associated to the PPD in C2C12 cells, we performed a computational fluxomic analysis. To this aim we used in-house developed software (Selivanov et al., 2006; Isodyn, Marin de Mas et al., 2011 and Supplementary Material 3). This software presents an excellent platform to integrate tracer-based metabolic data into a detailed computational model of the central carbon metabolism and enables to infer the metabolic flux profile that best explains the observed metabolic label distribution in both control and PPD-treated cells. Label distributions were integrated along with their concentrations in Isodyn. The results obtained with this software corroborated the previous results while enabled to have an overall view of the metabolic fluxes distribution (Figures 4A,B). As is described in Materials and Methods Section, we used the statistic χ^2^ to evaluate the reliability of our model predictions. Thus, the relatively low χ^2^-values obtained in this analysis in both control and PPD-treated cells (5.78 and 8.27, respectively, see Supplementary Material 1) indicate that the computed isotopologue fractions were consistent with the experimental measurements. The predicted isotopologue fractions (Figure 4A) are the result of a metabolic flux profile that in turn is defined by a set of kinetic parameters that minimize the difference between the calculated and measured isotopologue distribution (Supplementary Material 1). Thus, differences between groups in the calculated metabolic flux profile may define the metabolic processes underlying the aberrant metabolism associated to the exposure to PPD in C2C12 muscle cells. The calculated metabolic flux profile is depicted Figures 4A,B (see also Supplementary Material 1). Our model-driven analysis predicted a higher flux (relative to glucose uptake) through most of the glycolytic reaction in control group compared with PPD-treated cells, which indicates a higher glycolitic activity and is consistent with the higher label incorporation observe in lactate in control cells. Our computational analysis also predicted a higher glycogen turnover in control group which could explain the observed higher label incorporation. In addition, it was predicted a higher rate of xilulose-5-phosphate metabolization from fructose-6-phosphate in control group (through f6 → X5 reaction in Figures 4A,B) which is supported by a higher activity of the non-oxidative branch of ppp and the higher label incorporation to pentoses phosphate observed in control group that is discussed in previous sections. The lower pentose phosphate production predicted in PDD-treated cell may partially explain the decrease in the cell viability observed in C2C12 cell when are exposed to PPD. Finally, the model also predicted a high ATPase activity (atpase reaction in Figures 4A,B) in control group which is consistent with the higher cell viability that requires a more active energy metabolism.

## Discussion

PPD poisoning effects are characterized by vomiting and acute respiratory distress caused by a severe oedema of face, neck, pharynx, larynx, and upper airways. In a minority of the cases, hematuria, sometimes hemolysis and methaemoglobinuria, and acute renal failure also appear as late effects (Soni et al., 2009; Hooff et al., 2011). In the present study, animals treated with PPD at higher doses (40 and 60 mg/kg) presented the effects corresponding to both phases.

Although multiple factors can trigger rhabdomyolysis, it may be mediated by a final common pathway leading to muscle cell injury (muscle necrosis) and ultimately releasing cell components into the circulation (Melli et al., 2005). Our results show elevated levels of serum CK, LDH and aminotransferase enzymes (which are very specific markers for skeletal muscle injury) in Wistar rats treated with PPD. These findings are is in accordance with the primary diagnostic indicator of rhabdomyolysis.

Other cause of the rhabdomyolysis is the accumulation of sodium in the cytoplasm that leads to increase intracellular calcium concentration (Chavez et al., 2016). Here, calcium accumulation is mediated by several factors: (i) cell injury by itself, (ii) an enhanced Na^+^/Ca^2+^ exchange to compensate the excess sodium, and (iii) ATP depletion that reduce Ca^2+^-dependent ATPase activity and promotes Ca^2+^ accumulation (Hortelano Araque et al., 2008). Skinned fiber method has been applied to study the pharmacological effects of PPD on sarcoplasmic reticulum (SR) and contractile proteins in rat skeletal muscle fibers (Fajardo et al., 2013). Thus, it has been speculated that PPD can trigger rhabdomyolisys by promoting Ca^2+^ leakage from SR and calcium-induced calcium release (CICR), which lead to higher Ca^2+^ intracellular levels and promotescontinuous contraction and structural and metabolic changes in the muscle (Giulivi et al., 2011).

This fact can be related with the effect observed in animals treated with the higher doses of PPD, which showed hypoactivity, hypotonic, and ataxic gait during the treatment with this compound. In this sense our model-driven analysis predicted a significant reduction in the ATPase activity in C2C12 cells exposed to PPD. The lower flux through ATPase reaction is consistent with the biological observation previously discussed. In addition, it implies a reduced ATP production in PPD-treated cells which is also associated to rhabdomyolysis disease and may partially explain the higher cell viability observed in control cells that requires a more active energy metabolism.

The other important finding frequently observed in rhabdomyolysis is myoglobinuria. Myoglobinuria is always accompanied by rhabdomyolysis, but not necessarily the other way around. Urinary myoglobin provokes a typical reddish-brown (port-wine-like) color, even in absence of hematuria. Myoglobin is rapidly and unpredictably eliminated by hepatic metabolism (Wilson, 2006). After 24 h of treatment with PPD compound all animals had urine brown color but in absence of hematuria; nevertheless, 48 and 72 h after treatment this effect disappears. These facts could indicate that the effect of PPD is higher in early treatments because serum enzymes value gradually normalized and some symptoms disappeared, although the muscular weakness persisted for 3 days.

Furthermore, we studied how PPD-induced rhabdomyolysis affects central carbon metabolism in C2C12 muscle cell line. Thus, a first prospective approach applying metabolic Isotopomer Distribution Analysis (MIDA) showed significant differences in metabolic pathway patterns when C2C12 cells were treated with PPD to induce rhabdomyolysis. More specifically, we found that glucose was metabolized to lactate mainly through glycolysis in both treated and non-treated C2C12 cells. However, glycolytic contribution to lactate production is higher in non-treated C2C12 cells. The low label incorporation observed in Glutamate suggest a reduced activity of TCA cycle in C2C12 cells, disruption of glyclolysis by PPD may provoke ATP depletion, leading to Ca+ accumulation related with rhabdomyolysis due to consequent low activity of ATP-depending Ca channel. On the other hand, LDH liberation after PPD treatment may explain glycolytic inhibition, since LDH is needed to support glycolysis by recycling produced NADH to NAD^+^.

Additionally, differential changes in pentose phostphate pathways have been also observed. PPD cause a decrease in *de novo* synthesis of ribose-5-phosphate for nucleotide synthesis. The balance between non-oxidative and oxidative branches of pentose-phosphate pathway also decreased in PPD treated cells. Since oxidative branch of pentose-phosphate pathway is used not only for pentose phosphate synthesis but also for the generation of reduction power as NADPH, this decrease may affect cellular detoxification machinery of Reactive Oxygen Species (ROS; Dodson et al., 2013). Since ROS are well-known to trigger apoptosis (Halliwell, 2006), effects on ppp may correlate with effects on cell proliferation *in vitro* caused by these compounds, and specially, PPD inhibition of oxidative branch of ppp may explain enhanced apoptosis observed in treated cells (40% of apoptotic cells).

To further explore the metabolic effects of PPD-induced rhabdomyolysis we perform a metabolic flux analysis based kinetic modeling approach to analyze the tracer-based metabolic data of PPD-treated and control C2C12 cells. Our model-driven analysis permitted us to infer the metabolic flux alterations associated to PPD exposure. More specifically, our analysis suggests a higher glycolysis utilization compared with ppp activity in C2C12 cells which is in accordance with a higher enrichment of m2 lactate isotopologue (lactate generated from [1,2-^13^C_2_]-glucose via glycolysis) compared with m1 lactate isotopologue (lactate generated from [1,2-^13^C_2_]-glucose via glycolysis) in both cell groups. Furthermore, our model-driven analysis predicted a higher glycolytic activity relative to glucose consumption in control group that is consistent with the higher label incorporation observe in lactate in these cells. Our analysis also predicted a higher flux through the transketolase reaction that transforms fructorse-6-phosphate to xilulose5-phosphate in non-treated cells that is consistent with higher label enrichment in ribose-5-phosphate in control cells and with the MIDA analysis concluding a higher activity in the non-oxidative branch of ppp in control cells. Finally, the analysis determined a decrease in the flux through ATPase reaction leading to a reduction in ATP synthesis in treated cells. Gathering these evidences we have an overview in which PPD reduces ribose and ATP production which is consistent with the reduction in cell viability observed in PPD-treated C2C12 cells an in Wistar Rats and is supported by the experimental evidences.

In conclusion, the present study demonstrates that PPD induces some pathologic signs involved in rhabdomyolysis such as muscle necrosis, release of muscle components into the circulation, myoglobinuria, and muscle injury. Moreover, the present study has proven the potential of stable isotope techniques to understand the effects of PPD on central carbon metabolism. Our model-driven analysis has permitted to quantify the intracellular metabolic fluxes and the changes associated to PPD exposure which enabled a more in-depth understanding of the metabolic processes underlying the adverse effects associated with rhabdomyolysis on the C2C12 muscle cells. Thus, our work sheds new light on muscle dysfunction associated to PPD, opening new avenues for cost-effective therapies in Rhabdomyolysis disease.

## Author contributions

IM: Conception and design, assembly of data, data analysis, computational simulations, and interpretation and manuscript writing. SM and GP: Collection and assembly of data, interpretation, and manuscript writing. AA, JR, RT, and PV: Data interpretation and manuscript writing. VS: Data analysis and interpretation and manuscript writing. AL: Data interpretation and manuscript writing. MC: Conception and design, data analysis and interpretation, manuscript writing, and final approval of manuscript. All authors read and approved the final version of the manuscript.

### Conflict of interest statement

The authors declare that the research was conducted in the absence of any commercial or financial relationships that could be construed as a potential conflict of interest.

## Supplementary material

Supplementary Material 1 **Tables containing: (i) Complete list of isotopologue fractions of Glucose, Glycogen, Lactate, Glutamate fraction C2–5 and C2–4, and Ribose-5-phosphate of treated and non-treated C2C12 cells, (ii) Model-driven fitting of metabolite isotopologues and the corresponding χ^2^ of treated and non-treated C2C12 cells, (iii) kinetic parameters determined by our model-driven analysis and corresponding to the best fit solution, (iv) absolute metabolic fluxes determined by our model-driven analysis and corresponding to the best fit solution, and (v) metabolic fluxes relative to hexokinase flux, determined by our model-driven analysis and corresponding to the best fit solution**.

Supplementary Material 2 **Detailed description of Isodyn software**.

Supplementary Material 3 **Zip file with: (i) The kinetic model used to determine the metabolic fluxes, (ii) metabolic fluxes calculated for treated and non-treated C2C12 cells, (iii) kinetic parameters defined for treated and non-treated C2C12 cells, and (iv) results of the experimental labeling data for treated and non-treated C2C12 cells**.
