# Supplementary material for: Unveiling the Metabolic Changes on Muscle Cell Metabolism Underlying p-Phenylenediamine Toxicity
Source: Front Mol Biosci. 2017 Mar 6;4:8. doi: 10.3389/fmolb.2017.00008 (PMC5338303; doi:10.3389/fmolb.2017.00008)
Supplement: Supplementary Material 2 — Detailed description of Isodyn software. [file Presentation1.PDF]

Tracer-based metabolic flux analysis using Isodyn software

The computer program “Isodyn” (from “isotopomer dynamics”), which we developed in C++, represents a simulation environment for the dynamics of metabolite labeling by ^{13}C isotopes in metabolic reactions of living cells. For such simulations it uses a classical kinetic model of metabolic pathways linked with a module that computes the distribution of ^{13}C isotopic isomers of metabolites. Isodyn simulates ^{13}C isotopomer distribution in the same way as classical kinetic models simulate the time-course of metabolite concentrations. In its current version the software simulates the distribution of labeled carbons in the metabolites of metabolic pathways shown below.

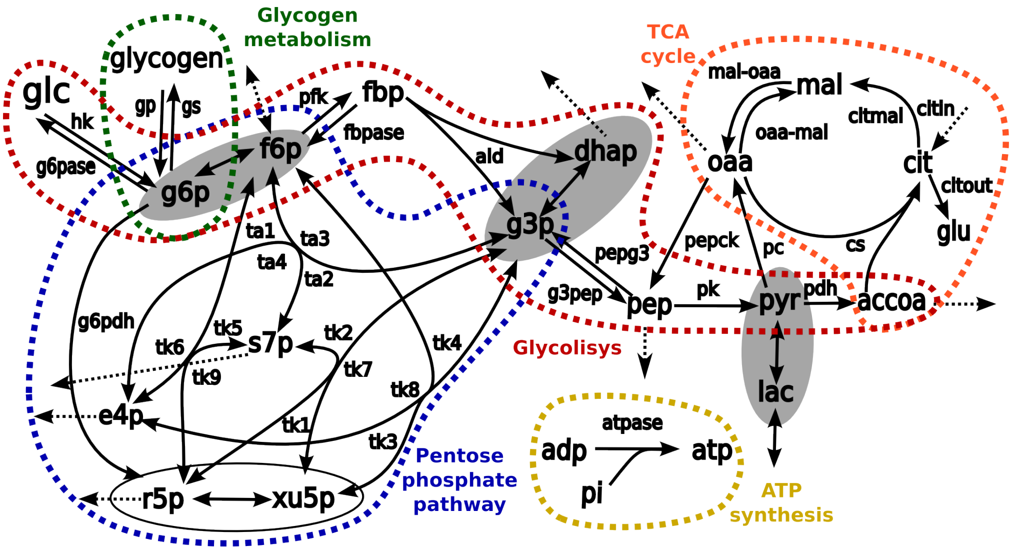

Figure 1. Scheme of pathways modeled in the current version of Isodyn.

Simulations are carried out by using a classical kinetic model of metabolic pathways linked with a module that computes the distribution of 13 C isotopic isomers of metabolites. Isodyn simulates ^{13}C isotopomer distributions in the same way as classical kinetic models simulate the time-course of metabolite concentrations [1-3].

The simulation of isotopomer distribution starts from the simulation of evolution of metabolic fluxes and concentrations in accordance with the scheme of metabolic pathways corresponding to the chosen set of parameters and initial concentrations. Such simulation is performed using the kinetic model, i.e. a system of ordinary differential equations (ODE) corresponding to the analyzed scheme of metabolic pathway. Numerical solution of such ODE system gives the time course of evolution of metabolite concentrations and fluxes. If metabolic steady state is simulated, the basic kinetic simulation lasts until the metabolic steady state is reached. After this simulation are calculated some additional isotope-exchange fluxes that are not needed for the kinetic model, but that are essential for the distribution of labeled carbons.

Then the total metabolite concentrations and the whole set of isotope exchange fluxes calculated in the previous steps are used in the module, which simulates the distribution of labeled carbons. This module automatically constructs hundreds of ODE describing the evolution of all isotopomers of the system. All these parts of the software are described in more details next:

Kinetic Model

The classical kinetic model is represented by a system of ordinary differential equations (ODE) simulating the evolution of concentra tions of metabolites:

$$\frac{dc}{dt} = \mathrm{Nv}\left(c, p\right) \quad (1)$$

Where here c = c1, c2, ..., cm is the vector of m metabolite concentrations described by the model, the metabolites considered in this particular case are presented in Figure 1.
N = nij, i = 1, . . . , m, j = 1, . . . , k is stoichiometric matrix for m metabolites and k reactions and v(c,p)=v1(c,p), v2(c,p), ...,vk(c,p), is the vector of k reaction rates (metabolic fluxes), which are functions of concentrations and parameters such as Vmax. Concentrations vary with time in accordance with (1), and parameters are constant in each simulation, but they are unknown and could be found by fitting the experimental data. The rate equations for the enzymatic reactions are in accordance with Michaelis-Menten mechanism among other kinetic mechanisms. Matrix N is organized in such a way that production (input) and consumption (output) for the metabolites corresponds to the arrows shown in Figure 1. By solving these equations numerically, the program computes evolution of total metabolite concentrations and metabolic fluxes for a given set of parameters (Vmax, Km, rate constants).
The common objective of ^{13}C metabolic flux analysis is the determination of metabolic fluxes that underly the measured distribution of isotopomers. This objective can be reached by the simulation of experimental data and determination of the best-fit set of fluxes. Since experimental data is the distribution of isotopomers, model must simulate it. According to (1) differential equation for a metabolite s is:

$$\frac{dC_S}{dt} = \sum n_{Sj} * v_j(c, p) \quad (2)$$

The set of parameters and metabolites concentrations values are specified in a text file ("nv.txt"), which could be changed externally and which the progran reads at the start of execution. The parameters and variables defined in nv.txt are used to determine metabolic fluxes by Michaelis-Menten equation or other kind of functions.

Isotopomerical model

The reactions j in (2), which change concentrations c_s, change also the concentrations of isotopomers $x_s = x_{s1}, x_{s2}, \ldots, x_{si}$. However, the net reaction rate vi accounted for kinetic model (1) could be composed of several isotope exchange processes, which differently affect isotopomer distribution and have to be accounted separately. Thus, reactions v_i should be decomposed to several reactions u_i:

$$u_i \rightarrow u_{i1}, u_{i2}, \ldots \quad (3)$$

This decomposition depends on reaction mechanism and is specific for each particular reaction [3].

Simulation of isotopomer distributions

After the decomposition (3), the differential equations for isotopomers of each of m considered metabolites could be presented in the form similar to (2):

$$\frac{dx_{Si}}{dt} = \sum_j \sum_h n_{sj} * w_j * (u_{jh}(c, p), c, x) \quad (4)$$

Here w_j is individual reaction rate that changes the concentration of isotopomer x_{sj}, which depends on the decomposition u_{jh} of rate v_j, vector of total concentrations c and isotopomer concentrations x. Equation (4) describes the evolution of concentration of isotopomer I of substance s. To simulate the isotopomers distribution in metabolites, which are described by kinetic model (1), the equations of type (4) must be written for all isotopomers of considered metabolites. This procedure is automated in Isodyn.
In general, large system for isotopomers (4) depends on and could be solved simultaneously with (1). However, if the dynamics of isotopomer distribution is simulated in the conditions of metabolic steady state, the procedure of numerical solution could be simplified as follows:

- System (1) could be solved independently from (4). The solution of system (1) gives steady state values of metabolic fluxes v i and concentrations c_i, which then could be used in (4) to evaluate the respective dynamics of isotopomer concentrations.

- Before solving the system (4) the net reaction rates v i obtained by solving (1) must be decomposed to the isotope exchange fluxes, which are specific for reaction mechanisms. For instance, if a reaction does not produce any change in carbon skeleton of substrates, the decomposition implies only those forward and reverse rates: $v_i \rightarrow u_f - u_r$. If a reaction performs splitting/reformation of carbon skeleton of substrate molecule, additional isotope-exchange fluxes, different from forward and reverse reaction rates, could take place.

The algorithms for the calculations of such isotope exchange fluxes for given parameters and metabolite concentrations are described elsewhere [2]. All the isotope exchange fluxes necessary for the calculation of isotopomer dynamics could be evaluated if the total metabolite concentrations are known after the execution of kinetic model. When the kinetic simulation is done, Isodyn calculates the necessary isotope exchange fluxes in one step, as explained in more details in Selivanov et al, 2005 [2].

When all the preparations are done (i.e. total metabolite concentrations and necessary fluxes are computed), the system of equations of type (4) for all isotopomer could be solved. Isodyn calls a module, which performs the computation of isotopomer dynamics.

The simulation of reaction mechanism is based on operations with binary numbers. Since only two carbon isotopes are considered, any combination of carbons in the skeleton of a molecule could be reflected by a respective binary number, where “1” states for ^{13}C and “0” states for ^{12}C. In this way all hexose isotopomers could be represented by numbers from 000000 (decimal 0) to 111111 (binary equivalent of decimal 63), triose isotopomers range from 000 to 111 (decimal 7), etc.

When Isodyn simulates reactions for isotopomers it splits-recombines the binary representation of isotopomers in the same way as enzymes split and reform molecules. For instance since aldolase splits hexose (fbp) into two trioses, the respective Isodyn function splits binary numbers of fbp representatives, taking them one by one subsequently, e.g. when it takes “010101”, it splits it to “010” and “101”. The number 010101 (binary equivalent of decimal 21) indicates the position of the value of this isotopomer concentration (C_{010101} with binary index or C_{21} with decimal index) in the array of concentrations of fbp isotopomers. So, it can calculate the reaction rate for a given isotopomer in a given reaction based on the total reaction rate fbp (u^{ald}_t) and total concentration (C^{ald}_t) known from the kinetic model simulation:

$$u_{21}^{ald} = \frac{u_t^{ald} * C_{21}}{C_t^{fbp}} \quad (5)$$

This rate is subtracted from the derivative of 21st isotopomer of fbp and added to the derivatives of trioses “010” (decimal 2) and “101” (decimal 5). The arrays of derivatives are organized in the same way as those for concentrations.

The same isotopomers could participate in various reactions. Isodyn simulates all of them adding the reaction rates (with correct sign) to the respective derivatives. The functions performing such simulations are described in supplementary information. They constitute a library, which can be used selectively.

Numerical solution

After the simulation in such a way of all the reactions of considered pathway, the whole array of derivatives for all isotopomers at a given time point is formed. The function that calculates derivatives as described above could be called by any ODE solver, which solves the ODE system thus constructed.

Isodyn implements several methods for ODE solving provided for C++ by Press et al [5], including fourth-order Runge-Kutta, Bulirsch-Stoer and Rosenbrock method for stiff systems. Also is implemented implicit Runge-Kutta 5th order method for stiff systems (Radau5), described in [6] and backward differentiation formulas as their implemented in the solver DASSL [7] written in Fortran but linked with the C++ code of Isodyn. The implementation of various methods allows finding the fastest solution for a given problem.

In the problem considered here the combination of DASSL for kinetic model with Runge-Kutta method as it is implemented in [5] for isotopomer distribution provided the fastest way to obtain solution.
The fact that solving the equations of kinetic model is followed by solving the system for isotopomers gives a way of checking solutions. Isodyn checks if the sum of isotopomers obtained after solving large isotopomer system is the same as total concentrations of metabolites obtained by kinetic model.
The large isotopomer system could easily become stiff so that the employed method of solution does not provide necessary accuracy. In this case the program indicates the inaccuracy of solution.

Fitting procedure
Simulation with an initial set of kinetic parameters gives a set of fluxes and corresponding isotopomer distribution, which could be compared with the measured distribution. Mass isotopomers of lactate and ribose were measured; to compare them with model prediction, the sums corresponding to respective measured mass isotopomers were calculated and the fractions with respect to the total amount were found. The difference between experimental data and the prediction was characterized by normalized square deviations:

$$\chi^2 = \sum_i \left[\frac{f_i^e - f_i^t}{\sigma_i^e} \right]^2 \quad (6)$$

Where f_i^e is experimental mass isotopomer fraction, f_i^t is the predicted one, σ_i^e is experimental standard deviation.
The first objective was to find the set of parameters (V_{max} for simple reactions described by Michaelis' equation with fixed Km).
To this end we modified Simulated Annealing algorithm [8], combining random change of parameter values (varying the amplitude of change like in Simulated Annealing) with Powell's method of coordinate descent [5], modifying each parameter in the direction that provides decrease of χ^2.
After each stochastic perturbation of parameters and descent to a local minimum of χ^2, Isodyn saved the set of parameters and respective fluxes corresponding to the local minimum. After performing several thousand perturbations and descents, the set corresponding to the absolute minimum among the obtained local minima was chosen as a best-fit set, and a range of fluxes corresponding to a specific level of confidence of χ^2, was taken as a confidence interval corresponding to the chosen level of confidence [5]. The procedure of fitting is schematically presented in Figure 2.

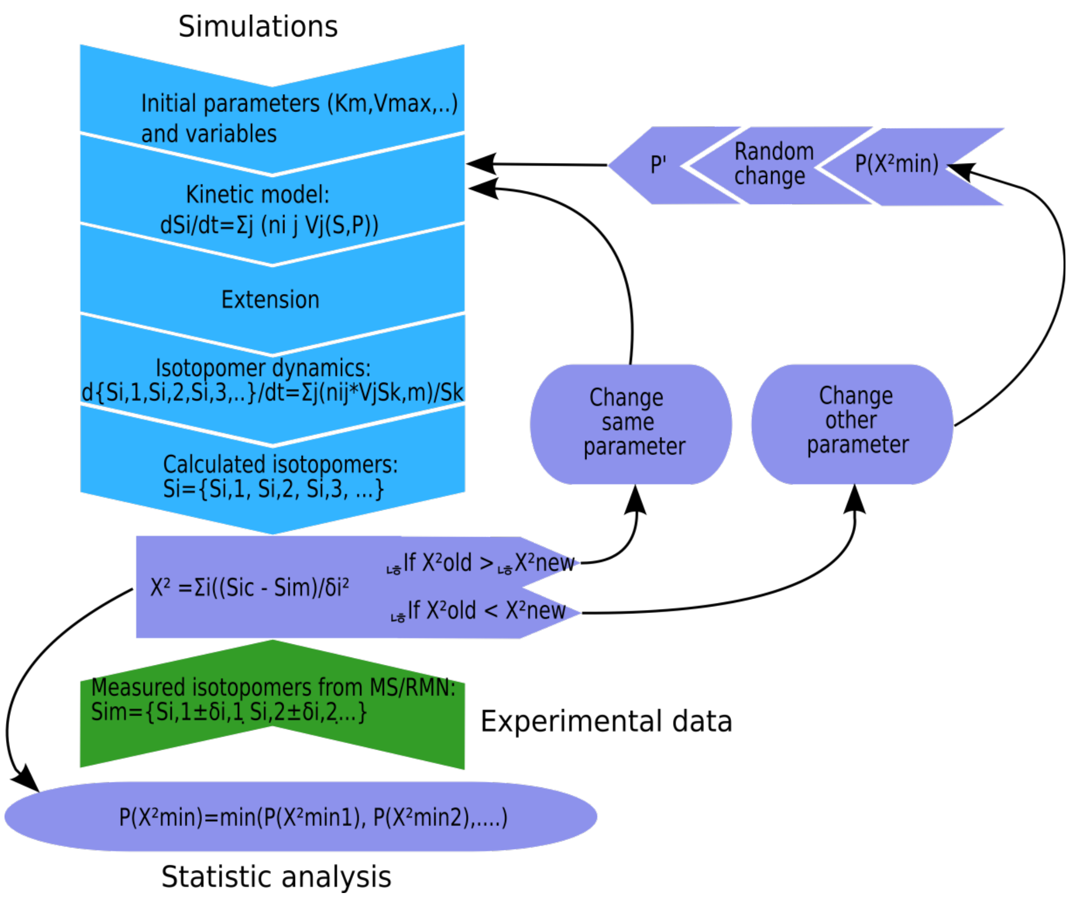

Figure 2.Scheme of parameter optimization in Isodyn.

Confidence intervals for metabolic fluxes

Constant χ^2 boundaries were used to define confidence limits as it is described in [5]. Before to use χ 2 boundaries for the estimation of confidence limits, the bestfit χ^2 value must be obtained.
Classical procedure is the following. Starting from the bestfit set of parameters we change one parameter and fix the changed value. Then fitting procedure is repeated with the chosen parameter fixed. Fixing one parameter at the value different from bestfit clearly will give higher χ^2 value. In case of normal distribution of measurement error the limits of parameter change, within which χ^2 increases not more that by 1.0, indicate 68% of confidence that the values within these limits include the “true” value. The range of the fixed parameter, where χ^2 increases not more than by 2.71 indicate 90% confidence limits, the increase by 4.0 indicate 95.4% confidence limits, 6.63 indicate 99% confidence limits.
Our procedure of finding the confidence limits was slightly different. The program repeated many times the fitting, starting from random set of parameters and each time finding local minimum and one global minimum corresponding to the best fit. Now, each local minimum could be considered as a result of fixing one arbitrary parameter at a value different from best fit and performing fitting with other parameters released. Proceeding in this way, all local minimums with χ^2 less than the considered confidence χ^2 limits must be collected, and the multitude of different values of each parameter taken separately could be considered as values that are within the chosen confidence interval for that parameter.
Since the metabolic fluxes are functions of the parameters, the same reasoning could be applied for the fluxes. The metabolic fluxes from the local χ^2 minimums that are within the selected χ^2 limit are within the chosen confidence limit.
This process is done for each flux and for each confidence boundary obtaining a table of fluxes with different confidence limits for different confidence boundaries.

Using Isodyn

As is exposed in previous sections the inputs of the tracer-based metabolic flux analysis with Isodyn are: i) a set of initial kinetic parameters to start the iteration process and ii) the experimentally measured metabolic isotopologue fractions, necessary for the fitting process. The initial kinetic parameters and experimental data are stored in the text files “nv.txt” and “expert.dat” respectively. On the other hand the results of the analysis are stored in different text files that are automatically generated by Isodyn along the computational process: i) in “dial.txt” file are stored the experimental and calculated isotopologue fractions with their associated χ^2 values, ii) in “statfl.csv” are stored the metabolic flux profile of the best fit analysis and iii) in “statfl.csv” the software save the kinetic parameters of the best fit analysis.
Isodyn is developed in C++ and run under Unix environment. To start the analysis, the executable file “a.out” must be invoked through the “./a.out” command

Supplementary references

1. Selivanov VA, Puigjaner J, Sillero A, Centelles JJ, Ramos-Montoya A, Lee PW, Cascante M: An optimized algorithm for flux estimation from isotopomer distribution in glucose metabolites. Bioinformatics 2004, 20:3387-3397.
2. Selivanov VA, Meshalkina LE, Solovjeva ON, Kuchel PW, Ramos-Montoya A, Kochetov GA, Lee PWN, Cascante M: Rapid simulation and analysis of isotopomer distributions using constraints based on enzyme mechanisms: an example from HT29 cancer cells. Bioinformatics 2005, 21:3558-3564.
3. Selivanov VA, Marin S, Lee PWN, Cascante M: Software for dynamic analysis of tracer-based metabolomic data: estimation of metabolic fluxes and their statistical analysis. Bioinformatics 2006, 22:2806-2812.
4. Marin de Mas I, Selivanov VA, Marin S, Roca J, Orešič M, Agius L, Cascante M. 2011. Compartmentation of glycogen metabolism revealed from 13C isotopologue distributions. *BMC Syst Biol.* **5**:175.
5. Press W, Flannery B, Teukolsky S, Vetterling W: Numerical Recipes in C: The Art of Scientific Computing Cambridge University Press, New York, USA 2002, 1:1-2.
6. Hairer E, Wanner G: Solving Ordinary Differential Equations II. Stiff and Differential-Algebraic Problems Springer Series in Computational Mathematics 14, Springer-Verlag 1996, 1:1.
7. Petzold LR: A description of DASSL: A differential/algebraic system solver Scientific Computing, eds RS Stepleman et al, North- Holland, Amsterdam 1983, 1:65-68.
8. Kirkpatrick S, Gelatt CD, Vecchi MP: Optimization by Simulated Annealing. Science 1983, 220:671-680.
