# Supplementary material for: Unveiling the Metabolic Changes on Muscle Cell Metabolism Underlying p-Phenylenediamine Toxicity
Source: Front Mol Biosci. 2017 Mar 6;4:8. doi: 10.3389/fmolb.2017.00008 (PMC5338303; doi:10.3389/fmolb.2017.00008)
Supplement: Supplementary Material 3 — Zip file with: (i) The kinetic model used to determine the metabolic fluxes, (ii) metabolic fluxes calculated for treated and non-treated C2C12 cells, (iii) kinetic parameters defined for treated and non-treated C2C12 cells, and (iv) results of the experimental labeling data for treated and non-treated C2C12 cells. [file Presentation2.ZIP › Marin de Mas et al_Manuscript_IM2.docx]

**Unveiling the metabolic changes on muscle cell metabolism underlying *p*-Phenylenediamine toxicity.**

Marín de Mas I.^1,2^, Marín S.^1^, Pachón G.^1^, Rodríguez-Prados JC.^1^, Vizán P.^1^,

Tauler R.^2^, Azqueta A.^2^, Selivanov V.^1^, López de Ceraín A.^3^, Cascante M.^* 1^

^-^

^1^ Departament de Bioquímica i Biologia Molecular. Facultat de Biología. Universitat de Barcelona, Barcelona. Spain.

^2^ Department of Environmental Chemistry, Institute of Environmental Assessment and Water Research (IDAEA), Consejo Superior de Investigaciones Científicas (CSIC), Barcelona, 08034, Catalonia, Spain.

^3^ Departamento de Bromatología, Tecnología de Alimentos y Toxicología, Facultad de Farmacia, Universidad de Navarra, Pamplona. Spain.

** Corresponding author:*

MC: [martacascante@ub.edu](mailto:martacascante@ub.edu)

**ABSTRACT**

Rhabdomyolysis is a disorder characterized by acute damage of the sarcolemma of the skeletal muscle leading to release of potentially toxic muscle cell components into the circulation, most notably creatine phosphokinase (CK) and myoglobulin, and is frequently accompanied by myoglobinuria.

In the present work, we tested the toxicity of *p*-phenylenediamine (PPD), a main component of hair dyes which is reported to induce rhabdomyolysis. We studied the metabolic effect of this compound *in vivo* with Wistar rats and in vitro with C2C12 muscle cells. To this aim we have combined multi-omic experimental measurements with computational approaches using model-driven methods. The integrative study presented here has unveiled the metabolic disorders associated to PPD exposure that may underlay the aberrant metabolism observed in rhabdomyolys disease.

Animals treated with lower doses of PPD (10mg/kg and 20mg/kg) showed depressed activity and myoglobinuria after 10 hours of treatment. Serum levels of aspartate aminotransferase (AST), alanine aminotransferase (ALT) and creatine kinase (CK) in rats treated 24, 48 and 72 hours were tested. At all times, treatment with PPD at higher doses (40 mg/kg and 60 mg/kg) showed an increase of AST and ALT, and also an increase of LDH and CK after 24 hours. Blood packed cell volume and haemoglobin levels, as well as organs weight at 48 and 72 hours, were also measured. No significant differences were observed in these parameters under any condition.

PPD induce cell cycle arrest in S phase and apoptosis (40% or early apoptotic cells) on *mus musculus* mouse C2C12 cells after 24h of treatment. Incubation of *mus musculus* mouse C2C12 cells with [1,2-^13^C_2_]-glucose during 24 h, subsequent quantification of ^13^C isotopologues distribution in key metabolites of glucose metabolic network and a computational fluxomic analysis using in-house developed software (Isodyn) showed that PPD is inhibiting glycolysis, non-oxidative pentose phosphate pathway, glycogen turnover and ATPAse reaction leading to a reduction in ATP synthesis. These predictions of the flux model unveil glucose metabolic network collapse consistent with the decrease in cell viability observed in PPD-treated C2C12 cells and with the myoglubinuria and other effects observed in Wistar Rats treated with PPD. These findings shed new light on muscle dysfunction associated to PPD exposure, opening new avenues for cost-effective therapies in Rhabdomyolysis disease.

**KEYWORDS**

Tracer-based metabolic data, Metabolic modeling, Metabolic Pathways, p-Phenylenediamine, Rhabdomyolysis

- - - - 1. INTRODUCTION

Rhabdomyolysis is a degenerative muscle disease, whose primary symptoms are muscle weakness, cramps, sweating and nausea, mainly because of muscle cells destruction (Melli *et al*., 2005; Wilson, 2006). Although the causes of rhabdomyolysis are diverse, current evidence suggests that there may be a common final pathway that mediates cellular injury. Thus some noxious factor, such as a drug that injures the plasma membrane of the cell, a toxin that activates a cytolytic enzyme, a factor that interferes with metabolism and disrupts the integrity of the skeletal muscle cell, a cytokine such as tumor necrosis factor, or simple hypoxia that reduces energy production by the cell, serves to increase cellular permeability to sodium ions. Then, sodium ions accumulation in the cytoplasm of the cell triggers an increase of cytosolic or mitochondrial calcium. Calcium activates a variety of proteolytic enzymes that injure the cell membrane, allowing efflux of cellular components into the circulation. Thus, some of these components, such as myoglobin or creatine kinase, facilitates clinical recognition of rhabdomyolysis (Melli *et al*., 2005).

A variety of studies report the capability of p-phenylenediamine (PPD) to induce skeletal muscle lesions in rats. PPD is one of the main components of hair dyes, which have been widely used to investigate the inflammatory and degenerative pattern of muscle fibers (Pourahmad *et al*., 2008; Garrigue *et al*., 2006; Zoll *et al*., 2015). PPD, together with several of its amino alkyl derivates are known to be myotoxic in animals and man, causing necrosis of cardiac and/or skeletal muscle (Iannotti *et al*., 2010). To clarify the mechanism of PPD related occurrence of rhabdomyolisys, we investigated the pharmacological effects of PPD on the function of the contractile proteins and the sarcoplasmic reticulum (SR) in single skeletal muscle fibers of the rat by using the skinned fiber method (Giulivi *et al*., 2011). Thus, it was speculated that PPD can bring out rhabdomyolisys by promoting the calcium-induced calcium release (CICR) and leakage of Ca^2+^ from the SR, being followed by an increase in the Ca^2+^ concentration and subsequent changes in the muscle, such as continuous contraction, an irreversible change in muscle structure and/or a hypermetabolic change (Giulivi *et al*., 2011).

PPD toxicity, and more specifically its role in rhabdomyolysis initiation, has been demonstrated by experiments conducted by administering PPD to mice (Chwaluk, 2013) that in turns alters creatine kinase (CK) levels in blood (Wilson, 2006).

In the present work, we assessed the toxicity of PPD, the antiproliferative effect on C2C12 cells, the capacity to induce cell cycle arrest and apoptosis and the molecular mechanisms triggered by this compound inducing rhabdiomyolysis in Wistar Rats.

Metabolism, as an effector of multiple cellular signals, represents the end point of many cellular events. Thus, metabolic characterization is a powerful tool to enhance our understanding of how compounds such as PPD exert their function on muscular cells *in vitro.* In this sense, the use of tracer-based metabolic substrates (i.e. [1,2-^13^C_2_]-glucose) combined with mass spectrometry (MS) permits to infer the metabolic flux through the main pathways associated with energy production and biosynthetic metabolism of the cell and has been revealed as a powerful tool to determine the metabolic profile of several *in vitro* models (Vizan *et al*., 2005; Boren *et al*., 2003; Kominsky *et al*., 2009; Burchert, 2007). More specifically, metabolic isotope distribution analysis (MIDA) or metabolic flux analysis via model-driven approaches permit the simulation and evaluation of substrate flux through major metabolic pathways under various physiological conditions (Cowan-Jacob *et al*., 2004).In order to correlate rhabdomyolysis and apoptosis associated to PPD with characteristic metabolic patterns, we modelled glucose metabolic network using available enzyme kinetic data, the experimentally measured ^13^C isotopomer distribution data and the previously developed software (Selivanov *et al*. 2006, Selivanov *et al*. 2005, Selivanov *et al*. 2004). This approach enabled evaluating the metabolic fluxe profiles in the analyzed metabolic network, which were supported by the mass isotopomers distribution obtained after incubation with labelled glucose in the presence of the PPD compound.

- - - - 1. Materials and methods

***In vivo* assays with Wistar Rats; Test animals and housing**

The study was approved by the Ethics Committee on Animal Experimentation of University of Navarra. Wistar female rats, with a body weight of 160 g ± 20% (8 weeks old), were supplied by Harlan (Harlan Laboratories, Indianapolis, IN). The animals were housed in polycarbonate cages with stainless steel covers in a room under controlled temperature conditions (22 ± 3 ºC), controlled relative humidity (50 ± 20%) and light (12 hours light–dark cycle). Air was exchanged 15times/h sterile food supplied by Harlan (Harlan Laboratories, Indianapolis, IN) and controlled water was available *ad libitum*. The rats were housed in the study room for 5 days prior to the start of treatment in order to allow acclimatization to the environmental conditions. Finally, we randomly assigned three rats to each studied group corresponding to different PPD doses (10, 20, 40 and 60 mg/Kg) and control (DMSO).

**Dosage and observations**

The animals were divided into groups of 3 rats at random. A control group receiving the vehicle and a second group receiving PPD at several concentrations 10mg/kg, 20mg/kg, 40mg/kg and 60mg/kg at single dose was included. The compound was administered in a volume of 300 µl of 100% DMSO by gavage. Animals were evaluated based on general appearance, behavior and signs of morbidity using the Irwin test (Castagné *et al*., 2012), immediately following administration, 30 minutes, 1, 2, 4 and 8 hours after the administration. All of the animals were weighed just before the acclimatization period, before administration and 24, 48 and 72 hours after administration. At the end of the observation period (24, 48 and 72 hours after administration), all the animals were anesthetized and blood samples were collected from tail and retro orbital sinus and centrifuged at 2000 xg for 30 min at 4ºC to recover the serum. The serum was frozen and stored at -20ºC until determination of biochemical parameters such as urea, creatinin, Glucose, aspartate aminotransferase (AST), alanine aminotransferase (ALT) and creatine kinase (CK), lactate dehydrogenase (LDH), at 24, 48 and 72 hours before sacrificed in a CO_2_ chamber and autopsied. All of the tissues were observed grossly. Ovaries, kidneys, liver, spleen, heart and lung were weighed and the relative organ weights were calculated. Serum CK and LDH levels were measured.

**Cell Culture**

We cultured *Mus musculus mouse C2C12* cells in DMEM, supplemented with 2mM glutamine, 10% heat-inactivated FCS, 10,000 units/ml of penicillin and 10 mg/ml of streptomycin. We used subconfluent monolayers of cells in all experiments. For biochemical characterization, C2C12 cells were cultured in DMEM, supplemented with 4mM asparagine without glutamine, 10% heat-inactivated FCS, 10,000 units/ml of penicillin and 10 mg/ml of streptomycin.

**MTT Assay**

We determined cell viability by measuring the absorbance of MTT dye staining of living cells (Pachon, *et al*., 2007; Pachon, *et al*., 2008).We cultured 3.5 x 10^3^ C2C12 cells/well in 96 well plates exposed to different concentrations of PPD concentrations ranging from 1μg/ml to 100μg/ml. We determined the relative cell viability by measuring the absorbance on an ELISA plate reader (Tecan Sunrise MR20-301, TECAN, Austria) at 550 nm. Concentrations that caused 50% (IC_50_) and 80% (IC_80_) of inhibition of C2C12 cells growth after 24 hours of treatment were calculated based on their survival rate compared with untreated cells.

**Cell cycle analysis**

We assessed cell cycle through flow cytometer by using a fluorescence-activated cell sorter (FACS) at 488 nm in an Epics XL flow cytometer (Coulter Corporation, Hialeah, FL, USA). This technique allows for the semiquantitation of DNA in each cell and permits to separate cells in G0 or G1 phase (one copy of DNA), G2 or M phase (after DNA duplication) and S phase (the amount of DNA per cell is in an intermediate state between the two previous conditions). Here we plated 4 x 10^5^ C2C12 cells/well in 6 well plates with 2ml of culture medium. After 24 hours of plated, PPD corresponding to IC_50_ and IC_80_ concentrations was added and cells were incubated for 24 hours, in parallel, control C2C12 cells were cultured without PPD. All experiments were performed five times with two replicates for each experiment.

**Annexin V-FITC binding assay**

This technique permits to determine whether a cell is at early or late apoptosis or necrotic state. In earlier apoptosis, Phosphatidylserine (PS) is translocated to outer membrane allowing PS-annexin V-FITC binding. In late apoptosis, as well as in necrotic processes, cell membranes lose their integrity allowing PI to access the nucleus and bind the DNA bases. Next, by using FACS analysis we can determine PI and annexin V-FITC staining accumulation to differentiate non-apoptotic cells (PI- and annexin V- ) from early apoptotic (PI- and annexin V+) and necrotic or late apoptotic (PI+) cells (Cortés et al., 2012). The same amount of C2C12 cells mentioned for cell cycle assay were treated with PPD as previously described. Cells were then collected and resuspended in binding buffer (10 mM Hepes/NaOH, pH7.4, 140 mM NaCl, 2.5 mM CaCl_2_). Annexin V-FITC conjugate (1μg/ml) was added and incubated for 30 minutes at room temperature in the dark. Just before FACS analysis (Bender medSystems (Innogenetics) human Annexin V-FITC Kit cat nº BMS306FI.), cells were stained with 1mg/ml PI solution. In each experiment approximately 20 x 10^3^ cells per condition were analyzed and the experiment was performed five times.

**Statistics**

Data is represented as the mean value from independent experiments ± SEM. For each assay, we used the Student’s *t* test for statistical analysis to compare the threated and the control untreated cells.

**Biochemical characterization of C2C12 cells**

We determined glucose and lactate concentration from culture medium byusing a Cobas Mira Plus chemistry analyzer (HORIBA ABX, Montpellier, France) at the beginning and at the end of incubation time (Cisternas et al., 2016).

**Metabolic profile of C2C12**

To track and measure the substrate carbon flux through central carbon metabolic pathways of C2C12 cells when treated with PPD, mass isotopomer distribution analysis (MIDA) and metabolic modeling approaches were used.

Sample preparation: Cells were cultured in DMEM (Biowithaker) supplemented with 10% dialyzed heat-inactivated FBS, glucose (10 mM, 50% isotope enrichment [1,2-^13^C_2_]-glucose), asparagine (20mg/ml), penicillin (100 U/ml) and streptomycin (100 µg/ml). PPD was added to the flasks using at the IC_50_ concentration determined for 24 hours (154 µM), in parallel, control C2C12 cells were cultured without PPD. Initially each flask contained 10^6^ cells and the incubation was carried out for 24 hours. At the end of incubations, we centrifuged cells (3000xg, 5 min) to obtain incubation medium and cell pellets. Afterwards, both pellet and medium were stored at -20 ºC for metabolite isolations.

Metabolite isolation and derivatization: We isolate and derivatized the following metabolites for their quantification in PPD-treated and non-treated C2C12 cells:

Glucose. To verify that 50% of glucose was [1,2-^13^C_2_] D-glucose, 500 μl of the initial medium were passed through a mixed DOWEX 50-DOWEX 1 ionic exchange column. Next, we passed 6 ml of milliQ water through the column and we collected the volume into a glass tube. Finally, we eliminated the remaining liquid by lyophilization for 12 hours and the dried sample was stored. We derivatized glucose to its aldonitrile acetate form by using hydroxyl-amine in pyridine and acetic anhydride. We monitored the ion cluster around the m/z 328 (C1–C6, chemical ionization) (Wamelink *et al*., 2008).

Glycogen. Glycogen for biochemical and isotopomerics distribution determination was extracted as described by Burchert(2007), by direct digestion of sonicated extracts with amyloglucosidase. Measurement of glucose released from glycogen was done by using the isotopomer [U-13C6-1,2,3,4,5,6,6-D7]D-glucose (or [U-13C-D7]-glucose as shorter name) as recovery standard and internal standard quantification procedures. The hydrolysed glucose was isolated using ion exchange chromatography (Mao *et al*., 2002) and was derivatized to its aldonitrile acetate form using hydroxyl-amine in pyridine and acetic anhydride. We monitored the ion cluster around the m/z 328 (C1–C6, chemical ionization) (Wamelink *et al*., 2008).

Lactate. Lactate from the culture media of C2C12 cells, which had been cultured and treated as explained above, was extracted by ethyl acetate. In more detail, 1ml of culture medium was acidified with 2-3 drops of HCl, followed by addition of 1ml of ethyl acetate and a little amount of sodium chloride and one minute shaken. After resting the samples for 2 minutes, we centrifugated them at 4,000 rpm for 10 minutes in a Jouan B3.11 centrifuge. We transfer the upper organic phase to another tube and to be subjected to slight N2 pressure for 10-15 minutes to remove the remaining ethyl acetate. We derivatized lactate to its propylamideheptafluorobutyric form and the m/z 328 (carbons 1–3 of lactate, chemical ionization) was monitored as described (Wamelink *et al*., 2008)*.*

RNA ribose. We isolated ribose from RNA by using acid hydrolysis of cellular RNA from the pellet of C2C12 treated and non-treated cells with the fractions as indicated above. We proceed as follows: i) firstly, we added 1 ml of Trizol to the cold pellets that were dissolved by sonication (30 seconds), ii) next, we transferred the homogenates to free RNase eppendorfs filled with 200 μl of chloroform. After shaking, we centrifuged the samples at 14,000 rpm for 30 minutes in an Eppendorf microfuge at 4ºC. We collected the chloroform and RNA from the non-colored upper phase, and was added 1 ml of cold 2-propanol. We precipitated the DNA by placing the mixure to repose overnight. Then, we centrifuged the samples under the conditions indicated above; we discarded supernatants and we added 1 ml of 75% cold ethanol to all of the pellets to further purify the RNA. Next, we shaked and centrifuged again to eliminated the supernatant. Were-suspended the in 10 μl of miliQ water and passed to a glass tube plus 2 ml of HCl 2N. Afterwards, we performed an acid hydrolysis of cellular RNA by heating the samples at 100 ºC for 2 hours followed by cooling at room temperature. Finally, we stored the tubes at - 80ºC or immediately dried to eliminate the remaining liquid by air pressure. We derivatized ribose isolated from RNA to its aldonitrile acetate form by using hydroxyl-amine in pyridine and acetic anhydride. We monitored the ion cluster around the m/z 256 (carbons 1–5 of ribose, chemical ionization) to determine the label 13C in ribose (Wamelink et *al.,* 2008).

Glutamate. We extracted glutamate from the culture media of C2C12 cells (cultured and treated as explained above) by using ammoniac and acetic acid. In more detail, 1ml of culture medium was passed through to DOWEX 50 ionic exchange column earlier prepared. Then, we passed 6 ml of milliQ water through the column and 5 ml of NH_4_OH 2N after, the volume was collected into a glass tube and immediately dried eliminating the remaining liquid by air pressure. Then, 6 ml of milliQ water were added to the tube and passed through to DOWEX 1 ionic exchange column earlier prepared and 5 ml of acetic acid CH_3_COOH 0.5N were added through the column. Finally, we collected the volume into a glass tube and eliminated the remaining liquid by air pressure for about 12 hours. We stored the dried sample until determination. Glutamate was converted to its nitrifluoroacetyl- n-butyl derivative and the ion clusters m/z 198 (carbons 2-5 of glutamate, electron impact ionization, EI) and m/z 152 (carbons 2-4 of glutamate, electron impact ionization, EI) were monitored. Isotopomeric analysis of C2-C5 and C2-C4 fragments of medium glutamate was performed in order to estimate the relative contributions of pyruvate carboxylase and pyruvate dehydrogenase to the tricarboxylic acid (TCA) cycle (Cowan-Jacob *et al*., 2004; Yoo *et al*., 2008).

Gas Chomatography/Mass Spectrometry (GC/MS). Mass spectral data were obtained on the HP5973 mass selective detector connected to an HP6890 gas chromatography. The settings were as follows: GC inlet 230ºC, transfer line 280ºC, MS source 230ºC, MS Quad 150ºC. An HP-5 capillary column (30m length, 250 µm diameter, 0,25 µm film thickness) was used for glucose, lactate, ribose, and glutamate analysis (Wamelink *et al*., 2008). A Bpx70 column (25-m length, 220-*µ*m diameter, 0.25- film thickness; SGE Incorporated, Austin, TX) was used for fatty acids analysis with specific temperature programming for each compound studied.

**Computational modeling of tracer-based metabolic data**

Transfer of ^13^C carbons from medium [1,2-^13^C2]-glucose into intracellular metabolites was simulated by using an in-house developed software for stable isotope tracer data analysis (Isodyn, Marin de Mas *et al*. 2011, Selivanov *et al*. 2006, Selivanov *et al*. 2005, Selivanov *et al*. 2004). The simulation scheme included all isotope-exchange reactions in pentose phosphate pathway, TCA cycle, glycolysis, anaplerotic reactions, exchange with extracellular glucose, lactate, and glutamate, ATP metabolism and also the biosynthetic fluxes (Figure 1). In brief, the software iteratively finds a set of parameters that defines metabolic flux profile to simulate the metabolic label distribution that best fits the experimental measurements. Finally, to evaluate the reliability of the analysis, χ² statistic is used to compare computed and measured label distributions as is described in equation 1. Here y_i_ is the experimental fraction measurement of the i^th^ isotope of metabolite y, y(x_i_,a) is the computed fraction of the same isotope and σ_i_ is the standard deviation of the i^th^ isotope of metabolite y.

$\chi^{2}=\sum_{i=1}^{N} \left[ \frac{y_{i}-y\left( x_{i};a \right)}{\sigma_{i}} \right]^{2}$ (1)

Data analysis using this software allowed us to integrate tracer-based metabolic data and metabolite consumption/production rates to infer the internal metabolic fluxes in control and PPD-treated cells. A more detailed description of Isodyn software is in the Supplementary material 2.


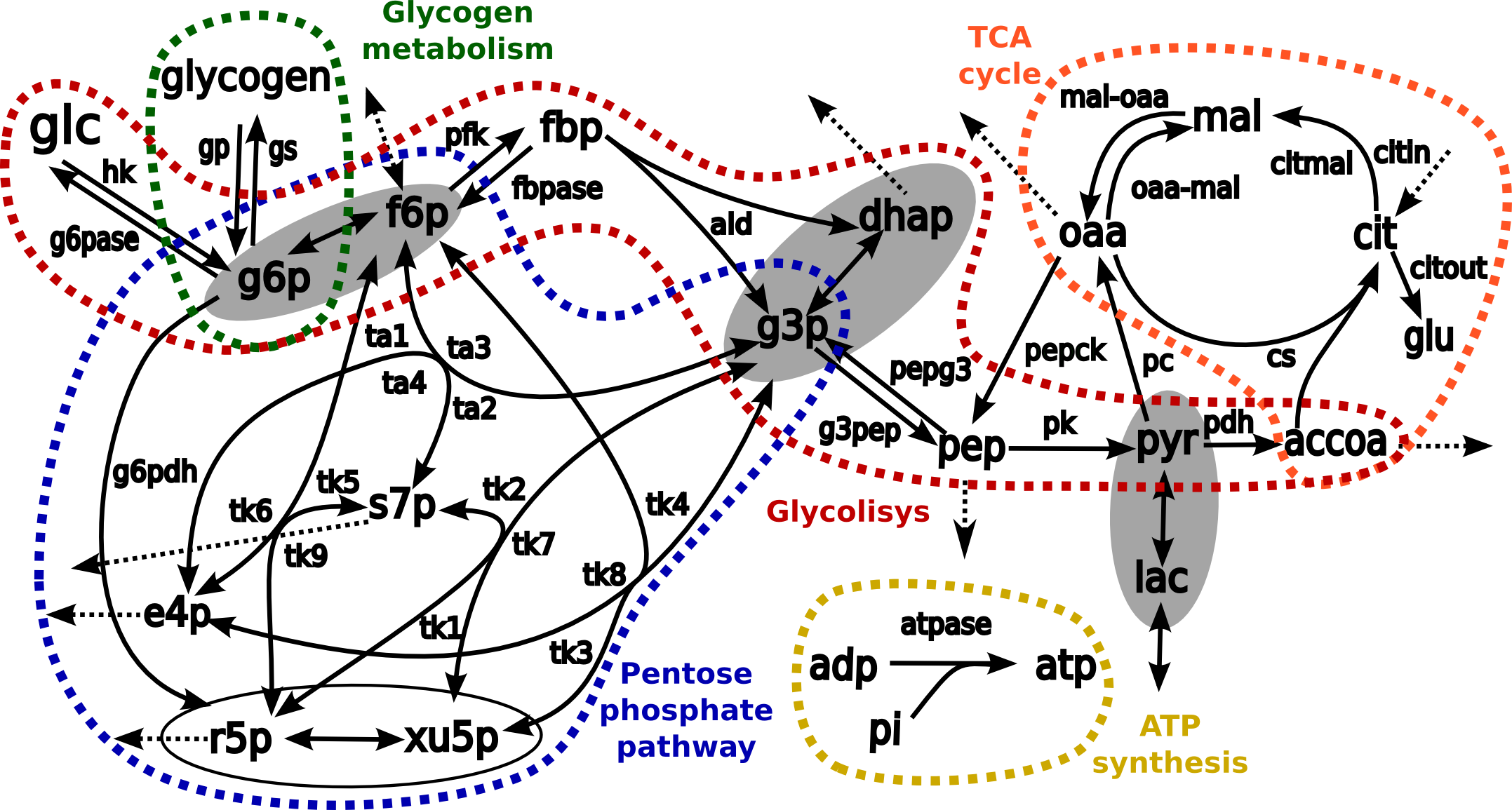


**Figure 1.** The schemes of kinetic model used as a base for simulation of isotopologue distribution. Metabolites are connected through biochemical reactions represented by arrows. Pathways considered in the model are enclosed and highlighted in different colours: red, glycolysis; blue, pentose phosphate pathways; orange, TCA cycle and green glycogen metabolism. The metabolites enclosed in ellipses are considered to be in fast equilibrium. List of abbreviations Metabolites: glc: glucose; glu: glutamate; lac: lactate; glgn: glycogen; g6p: glucose 6 phosphate; f6p: fructose 6-phosphate; fbp: fructose 1,6-bisphosphate; dhap: dihydroxyacetone phosphate; g3p: glyceraldehyde 3-phosphate; pep: phosphoenolpyruvate; pyr: pyruvate; accoa: acetyl coenzyme A; e4p: erythrose 4-phosphate; s7p: sedoheptulose 7-phosphate; r5p: ribose 5-phosphate; xu5p: xylulose 5-phosphate; mal: malate; oaa: oxaloacetic acid; cit: citrate; adp: adenosine diphosphate and atp: adenosine triphosphate . Enzymes: g6pase: glucose 6-phosphatase; gs: glycogen synthase; gp: glycogen phosphorylase; hk: hexokinase; fbp: fructose 1,6-bisphosphatase; pfk: phosphofructokinase; ald: aldolase; g6pdh: glucose 6-phosphate dehydrogenase; pepck: phosphoenolpyruvate carboxykinase; pk: pyruvate kinase; pdh: pyruvate dehydrogenase complex; cs: citrate synthase; pc: pyruvate carboxylase; atpase: ATP synthesis; pepg3: pathway pep→g3p; g3pep: pathway g3p→pep; mal-oaa: pathway mal→oaa; oaa-mal: pathway oaa→mal; transaldolase activity: ta1: f6p→s7p, ta2: s7p→ f6p, ta3: f6p↔g3p and ta4: s7p↔e4p; transketolase activity: tk1: x5p→s7p, tk2: s7p→x5p, tk3:f6p→x5p, tk4:x5p→f6p, tk5: f6p→s7p, tk6: s7p→f6p, tk7: x5p↔g3p, tk8: f6p↔e4p and tk9: r5p↔s7p.

**Data analysis and statistical methods**

*In vitro* experiments were carried out using three cultures for each treatment. Mass spectral analyses were carried out by three independent automatic injections by the sampler and accepted only if the standard sample deviation was less than 1% of the normalized peak intensity. Statistical analyses were performed by the non-parametric Mann-Whitney U test, with two independent samples. The p ≤ 0.05 was considered to indicate significant differences in glucose carbon metabolism between control and PPD.

**Results**

PPD presents toxic effects and alters the molecular biomarkers in blood on Wistar Rats.

Increasing doses of PPD (from 10 mg/Kg of weight body to 60 mg/kg of weight body) was administered to Wistar female rats. Immediately after administration, all of the animals presented a decrease in the spontaneous reactivity, which lasted between 10 hours and 2 days, depending on the doses (Table 1). These symptoms were not observed in the control animals. The evolution of body weight was normal in all groups. Changes in the general state, external appearance or behavior were observed along the observation period in animals treated with PPD and a general depressed activity and piloerection which lasted from 10 hours until death, depending on the doses (Table 1). Animals treated with PPD at lower doses (10mg/kg and 20mg/kg) until 10 hours of treatment, showed depressed activity and some of they had brown color urine or myoglobinuria. Animals treated with higher doses of PPD (40mg/kg and 60mg/kg) until 4 hours of treatment, showed total loss of equilibrium, hypotonic gait and piloerection. After 48 hours of treatment with PPD at 60mg/kg, mices they ataxic gait, but this effect disappeared after 72 hours of treatment. After 48 and 72 hours of treatment, one animal of each group of PPD treatment was sacrificed and necropsied

| Compound | Doses | Hipoactivity | Piloerection | Ataxic. G | Hypot. G | Myoglo |
| --- | --- | --- | --- | --- | --- | --- |
| PPD | 10 | 10 h | 2 - 4 h | 0/3 | 0/3 | ++ |
|  | 20 | 10 h | 2 - 4 h | 0/3 | 0/3 | ++ |
|  | 40 | 1 day | Until death | 2/3 | 0/3 |  |
|  | 60 | 2 days | Until death | 0/3 | 3/3 |  |
| DMSO | 300 µl | 30 min | 1 h | 0/3 | 0/3 |  |

**Table 1.** Toxicity after oral administration of four concentrations of PPD compound in mg/kg. The compound was dissolved in DMSO and 300 µl of solution was administered. Animals were sacrificed after 48 and 72h of observational period. Periods of time are in hours (h), days or minutes (min). ++ represents the presence of myoglobinuria.; Ataxic G: Ataxic Gait; Hypot G: Hypotonic Gait; Myoglo: Myoglobiunuria.

Biochemical parameters in blood were measured 24, 48 and 72 hours after PPD dosage. Results obtained with animals treated with PPD compound indicate that AST and ALT (Figure 2a) increased at 24, 48 and 72 hours, and LDH and CK enzymes (Figure 2a) increased at 24 hours after the final dose of treatment at higher doses of PPD (40 mg/kg and 60 mg/kg).

Blood packed cell volume and haemoglobin levels, as well as organs weight at 48 and 72 hours, were also measured. No significant differences were observed in these parameters under any condition. Histological analysis of the different organs extracted showed also similar patterns as controls.

Antiproliferative effect of PPD on C2C12 cells

The antiproliferative effect of PPD was investigated in C2C12 cells after 24 hours of incubation. The IC_50_ concentrations were obtained by MTT test. PPD concentrations were plotted against the percentage of cell proliferation after 24, 48 and 72 hours of incubation. The IC_50_ values obtained in C2C12 cells were 154 ± 6 μM, 108 ± 8 μM and 59 ± 1 μM at 24, 48 and 72 hours respectively and the IC_80_ values were 201 ± 8 μM, 182 ± 12 μM and 91 ± 7 μM at 24, 48 and 72 hours respectively (Figure 2b).

Cell Cycle and Apoptosis: C2C12 cells treated with PPD at IC_50_ and IC_80_ concentrations after 24 hours of treatment showed an increase in the population in S phase (13% at IC_50_, and 28% at IC_80_ for C2C12 cells respectively) compared to untreated cells. It was accompanied by a concomitant decrease in the percentage of cells in the G0/G1 phases (19% at IC_50_, and 32% at IC_80_ for C2C12 cells), suggesting an increase in the S-phase arrest (Figure 2c).

Apoptosis was assessed in C2C12 cells after 24 hours of treatment with PPD at the same concentrations mentioned above for the analysis of cell cycle. We differentiate early apoptotic cells (annexin V^+^ and PI^-^) from late apoptotic/necrotic cells (annexin V^+^ and PI^+^) by performing FACS analysis using annexin V-FITC staining and PI accumulation . Results showed that C2C12 cells treated with PPD at IC_50_ concentration increase apoptosis in 55% (34.7% and 18.8% of early and late apoptosis respectively) with respect to the untreated cells. We also observed that cells treated with PPD at IC_80_ concentration icreased apoptosis in 43% (20.1% early apoptosis plus 23.9 % late apoptosis) compared with non-treated C2C12 cells (Figure 2d). Annexin-V binding assay showed that PPD induced a high number of apoptotic cells.


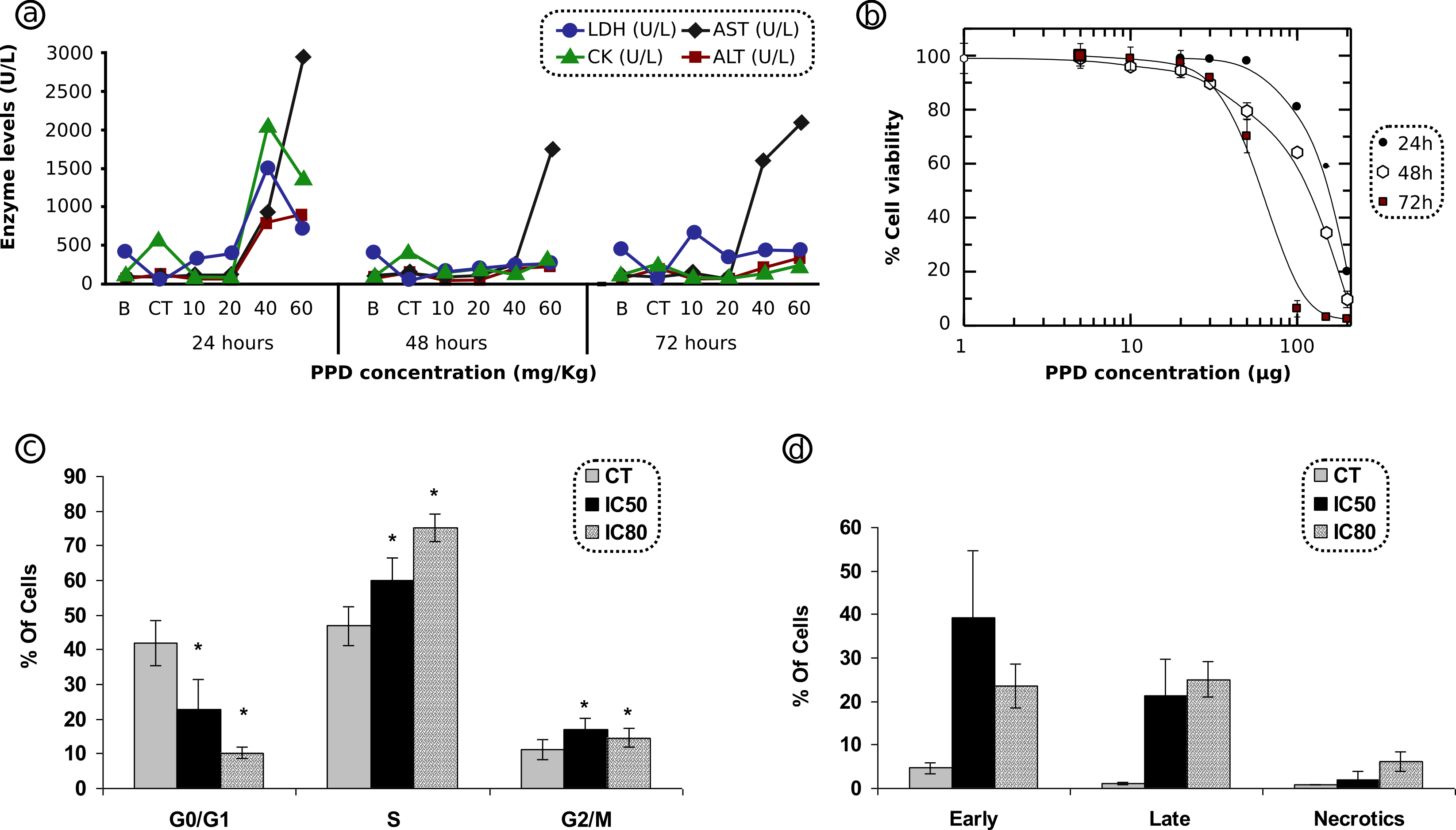


Figure 2. (a) LDH, CK, AST and ALT levels after 24, 48 and 72 hours of PPD treatment at different doses administration in Wistar rats, control group (CT) and the enzyme levels before PPD administration (B). The measurements are given in U/L (International unit of enzymatic activity per liter). (b) Effect of PPD doses (in µg) on cell proliferation of C2C12 cells, after 24, 48 and 72 hours of incubation. The relative percentage of cell proliferation was calculated assuming a 100% of proliferation in untreated cells at 24, 48 and 72 hours. Each point represents the mean of triplicate experiments. (c) Cell cycle analysis of PPD treated cells. C2C12 cells were treated for 24h at 37ºC with either IC_50_ or IC_80_ (second and third column respectively) concentrations, or the solvent ( first column as control). Cell cycle analysis was conducted after propidium iodide staining. Values are represented as mean ± SE of five independents experiments. (*), significantly different at p < 0.05 compared with control cells. (d) Flow cytometry analysis of propidium iodide accumulation and Annexin V-FITC staining and after exposure of C2C12 cells to IC_50_ and IC_80_ concentrations (second and third column respectively), or the solvent (first column as control) for 24 hours. Values are expressed as mean ± SD of five independents experiments.

Determining differences in central carbon metabolism activity in C2C12 cells treated with PPD via mass isotopomer distribution analysis (MIDA)

Glucose: Glucose consumption and lactate production were determined by using espectrophotometric methods following 24 hours of incubation of C2C12 cells treated with PPD at IC_50_. The results didn't show differences between the initial and the final ^13^C enrichment in glucose (46.3% of [1,2-^13^C_2_]-glucose, glucose m1 in figure 3.a -m0, non-labeled; m1, containing one ^13^C isotope; m2, two ^13^C isotopes, etc…), which means that no glucose was released.

**Lactate:** An approximate estimation of the metabolic flux through the pentose phosphate pathway (ppp) and glycolysis can be inferred by measuring the ^13^C label distribution in lactate (Lactate isotopologue distribution is depicted in figure 3.a). The two ^13^C labelled lactate (m2 in figure 3.a) is directly produced via glycolysis, whereas one ^13^C labelled lactate (m1 figure 3.a) is the product of the oxidation of [1,2-^13^C_2_]-glucose via oxidative branch of pentose-phosphate pathway and then recycled to glycolysis through the non-oxidative branch of pentose-phosphate pathway (Wamelink *et al*., 2008). Thus, the percentage of glucose metabolized directly to lactate can be determined by applying the formula: Glycolitic rate = [Δlactate × m2_lactate_] / [Δglucose × m2_glucose_], where Δglucose is the glucose consumption (Control: 2,31 ± 0,04 mM; PPD: 2,42 ± 0,26 mM.) and Δlactate is the lactate production (Control: 2,5 ± 1,0 mM; PPD: 1,5 ± 0,3 mM). Here we didn't find significant differences in the glycolitic rate between the PPD-treated and control cells (28,8 ± 14% and 31,9 ± 13%, respectively). Additionally, we did not observe significant enrichment in m1 lactate isotopomer which indicates a low activity of ppp and that glucose was metabolized to lactate mainly through glycolysis in both groups. The results also showed that glycolytic contribution to lactate production is higher in non-treated C2C12 cells.

Glycogen: We didn't observe significant differences in the total glycogen reservoirs in the C2C12 cells after 24 hours of incubation in none of the groups(10,1 µg of glycogen). Furthermore, significant ^13^C incorporation was observed in glycogen after incubation. More specifically glycogen was enriched in m2 species in both groups (glycogen m2 in figure 3.a), which suggests an important exchange between medium [1,2-^13^C_2_]-glucose, and glycogen reservoirs. On the other hand, m1 species were no significant in any of the groups (glycogen m1 in figure 3.a) which suggest a low contribution of ppp to glycogen metabolism in C2C12 cells and is supported by our previous analysis of glucose and lactate labeling data.

**Ribose:** To estimate the nucleic acid precursor synthesis in C2C12 cells treated with PPD, measurements of the molar enrichment of RNA ribose from [1,2-^13^C_2_]-glucose were carried out by calculating the overall ^13^C enrichment by applying the following formula: Σm_n_ = m1 + 2×m2 + 3×m3 + 4×m4 + 5×m5 . This calculation showed that Σm_n_ ribose label enrichment significantly decrease after treatment with PPD (0,294 ± 0,028 in control and 0,197 ± 0,023 in PPD-treated cells), which indicates an inhibition of *de novo* nucleic acid synthesis in treated cells in comparison with the control cells.

Additionally, we found significant differences in the balance between non-oxidative and oxidative branch of pentose-phosphate pathway between control and PPD-treated cells. It was achieved calculating the ratio between both branches of ppp as is described in Wamelink *et al*., 2008: ox/non-ox= m1+ m3/ (m2 + m3 + 2m4). Here we found that this ratio was 1,183 ± 0,004 in control cells and 2,224 ± 0,189 in PPD-treated cells. These differences are mainly due to the low activity of the non-oxidative branch of pentose-phosphate pathway in PPD-treated cells as the glucose consumption is drive to lactate production and no r5p is recycled to glycolysis. Besides, the activity of the oxidative branch of pentose-phosphate pathway is also lower in PPD treated cell allowing a lower molar enrichment in ribose.

Moreover, these results correlate with the fact that m1 lactate isotopomer was almost undetected (Supplementary material 1) in both groups, suggesting that in both conditions glycolysis is the preferred pathway to glucose consumption.

**Glutamate:** Glutamate, a non-essential amino-acid, is partially produced from mitochondrial α-ketoglutarate, which is a central intermediate of the TCA cycle. Therefore, label incorporation from glucose into glutamate is a good indicator of TCA cycle anabolic metabolism for amino acid synthesis instead of glucose oxidation (Wamelink *et al*., 2008). The analysis did not reveal significant ^13^C enrichment in glutamate in any of both C2C12 cells groups.

**Unveiling metabolic flux alterations in C2C12 cells treated with PPD via model-driven method**

In order to perform a more in-depth analysis of the tracer-based metabolic data and to identify the key metabolic adaptations associated to the PPD in C2C12 cells, we performed a computational fluxomic analysis. To this aim we used in-house developed software (Isodyn, Marin de Mas *et al* 2011, Selivanov *et al* 2006 and Supplementary Material 3). This software presents an excellent platform to integrate tracer-based metabolic data into a detailed computational model of the central carbon metabolism and enables to infer the metabolic flux profile that best explains the observed metabolic label distribution in both control and PPD-treated cells. Label distributions were integrated along with their concentrations in Isodyn. The results obtained with this software corroborated the previous results while enabled to have an overall view of the metabolic fluxes distribution (figure 3.a and 3.b). As is described in Methods section, we used the statistic Χ2 to evaluate the reliability of our model predictions. Thus, the relatively low Χ2 values obtained in this analysis in both control and PPD-treated cells (5.78 and 8.27 respectively, see Supplementary material 1) indicate that the computed isotopologue fractions are consistent with the experimental measurements. The predicted isotopologue fractions (figure 3.a) are the result of a metabolic flux profile that in turn is defined by a set of kinetic parameters that minimize the difference between the calculated and measured isotopologue distribution (Supplementary material 1). Thus, differences between groups in the calculated metabolic flux profile may define the metabolic processes underlying the aberrant metabolism associated to the exposure to PPD in C2C12 muscle cells. The calculated metabolic flux profile is depicted figure 3.a and 3.b (see also Supplementary material 1). Our model-driven analysis predicted a higher flux (relative to glucose uptake) through most of the glycolytic reaction in control group compared with PPD-treated cells, which indicates a higher glycolitic activity and is consistent with the higher label incorporation observe in lactate in control cells. Our computational analysis also predicted a higher glycogen turnover in control group which could explain the observed higher label incorporation. In addition, it was predicted a higher rate of xilulose-5-phosphate metabolization from fructose-6-phosphate in control group (through f6 → X5 reaction in figure 3.a and 3.b) which is supported by a higher activity of the non-oxidative branch of ppp and the higher label incorporation to pentoses phosphate observed in control group that is discussed in previous sections. The lower pentose phosphate production predicted in PDD-treated cell may partially explain the decrease in the cell viability observed in C2C12 cell when are exposed to PPD. Finally, the model also predicted a high ATPase activity (atpase reaction in figure 3.a and 3.b) in control group which is consistent with the higher cell viability that requires a more active energy metabolism.


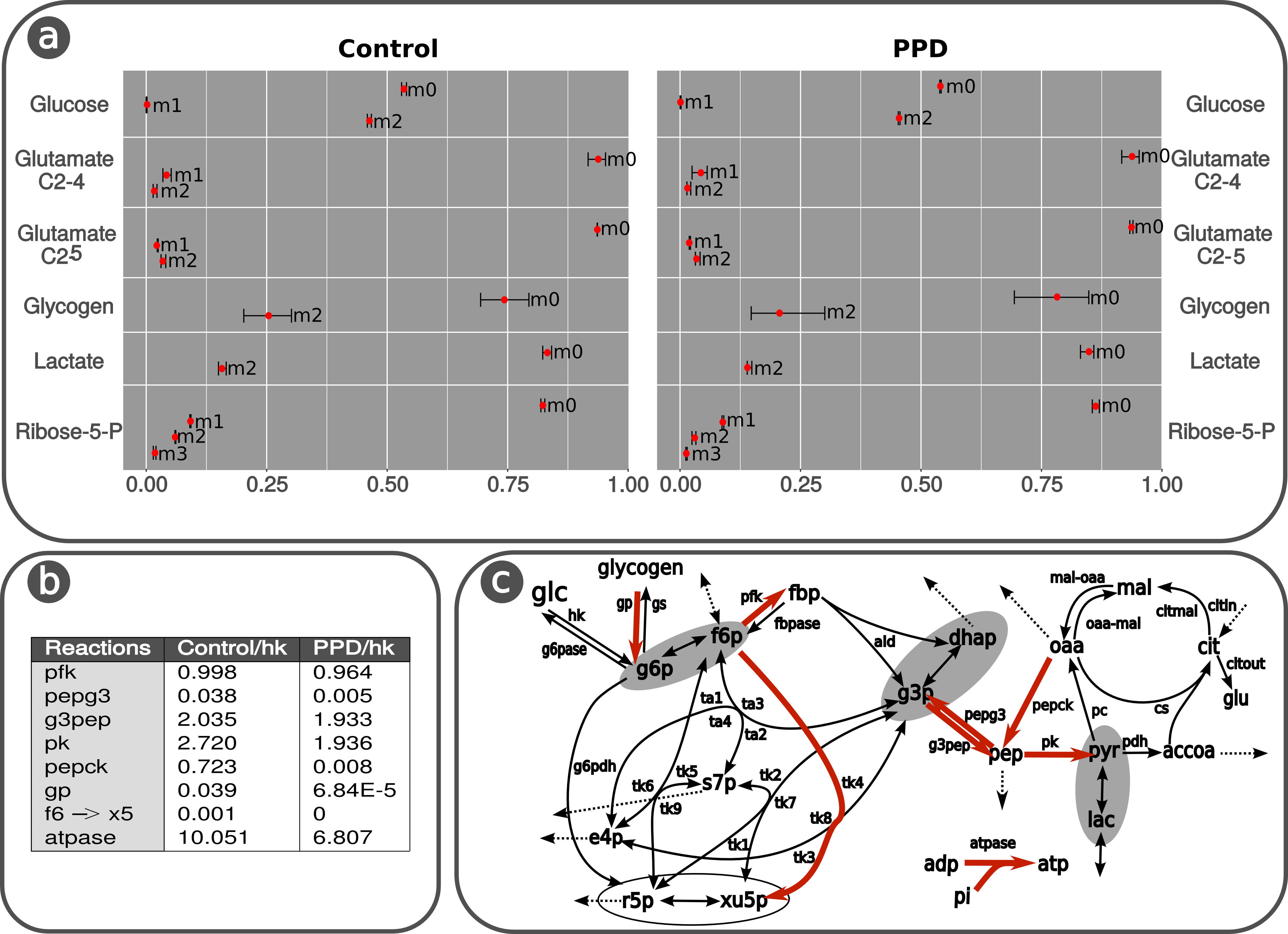


**Figure 3. a.** Isotopologues (m0, non-labeled; m1, containing one ^13^C isotope; m2, two ^13^C isotopes, etc) produced by isolated C2C12 cells from glucose as the only substrate contained 50% of [1,2-^13^C2]D-glucose were measured in glucose from medium, glucose from glycogen, lactate, and fragments of glutamate after 24 hours exposed to PPD and non-exposed (control). The measurements are presented as mean ± standard deviation and the predicted label distribution for each isotopologue is represented with red points. The data was simulated using a model in accordance with the scheme presented in Figure1. The fitting was performed using a stochastic algorithm described in Marin de Mas *et al*., 2011. The difference between the best fit and experimental data (Methods) are summarized for the whole set of data. The whole isotope distribution can be found in the Supplementary material 1. **b.** Principal fluxes of central carbon metabolism estimated in control and PPD-treated C2C12 cells. This table shows the fluxes with significant differences between groups (p<0.05). Fluxes are expressed as mmol×mL-1min-1×flux hk. Abbreviations used in this table correspond to flux notation in figure 1. The whole isotope distribution can be found in the Supplementary material 1. **c.** The schemes of kinetic model used as a base for simulation of isotopologue distribution. Principal fluxes of central carbon metabolism estimated in control and PPD-treated C2C12 cells. Metaboilc reactions highlighted in red represent predicted fluxes significantly higher in control cells (no reactions with higher flux in PPD-treated group were predicted). Abbreviations used in this figure correspond to flux notation in figure 1.

**Discussion**

The effect of PPD poisoning may be divided into two phases. The first appearing shortly after ingestion consist of vomiting and severe oedema of the face, neek, pharynkx, larynx and upper airways, culminating in acute respiratory distress. The second phase which appear later in a minority of the victims consists of haematuria, sometimes haemolysis and methaemoglobinuria, and acute renal failure (Hooff et al., 2011; Soni *et al*., 2009). In the present study, animals treated with PPD at higher doses (40mg/kg and 60mg/kg) presented the effects corresponding to both phases.

Although multiple factors may trigger rhabdomyolysis, the pathogenesis appears to follow a final common pathway, ultimately leading to muscle necrosis and release of muscle components into the circulation (Melli *et al*., 2005). Our results show elevated levels of serum CK, LDH and aminotransferase enzymes (which are very specific markers for skeletal muscle injury) in Wistar rats treated with PPD. These findings are is in accordance with the primary diagnostic indicator of rhabdomyolysis.

Other cause of the rhabdomyolysis is the accumulation of sodium in the cytoplasm that leads to increase intracellular calcium concentration (Chavez *et al*., 2016). This accumulation of calcium is due both to direct injury of the cell and to an increased activity of Na^+^/Ca^2+^ exchange protein that provoke calcium into the cells as it attempts to remove the excess sodium. Depletion of ATP also contributes directly to calcium accumulation due to a reduction in the activity of the Ca^2+^ ATPase, which normally acts to pump calcium out of the cell and accumulate it in the sarcoplasmic reticulum (Hortelano Araque *et al*., 2008). The pharmacological effects of PPD on the function of the contractile proteins and the sarcoplasmic reticulum (SR) have been investigated in single skeletal muscle fibers of the rat by using the skinned fiber method ([Fajardo](https://www.ncbi.nlm.nih.gov/pubmed/?term=Fajardo%20VA%5BAuthor%5D&cauthor=true&cauthor_uid=23430510) *et al.*, 2013). Thus, it has been speculated that PPD can trigger rhabdomyolisys by promoting the calcium-induced calcium release (CICR) and leakage of Ca2+ f^ro^m the SR, being followed by an increase in the Ca2+ c^on^centration and consequent changes in the muscle, such as continuous contraction, an irreversible change in muscle structure and/or a hypermetabolic change (Giulivi et a*l., 2*011). This fact can be related with the effect observed in animals treated with the higher doses of PPD, which showed hypoactivity, hypotonic and ataxic gait during the treatment with this compound. In this sense our model-driven analysis predicted a significant reduction in the ATPase activity in C2C12 cells exposed to PPD. The lower flux through ATPase reaction is consistent with the biological observation previously discussed. In addition, it implies a reduced ATP production in PPD-treated cells which is also associated to rhabdomyolysis disease and may partially explain the higher cell viability observed in control cells that requires a more active energy metabolism.

The other important finding frequently observed in rhabdomyolysis is myoglobinuria. Myoglobinuria does not occur without rhabdomyolysis, but rhabdomyolysis not necessarily results in visible myoglobinuria. Urinary myoglobin provokes a typical reddish-brown (port-wine-like) color, even in absence of hematuria. Myoglobin is rapidly and unpredictably eliminated by hepatic metabolism (Wilson, 2006). After 24 hours of treatment with PPD compound all animals had urine brown color but in absence of hematuria; nevertheless, 48 and 72 hour after treatment this effect disappears. These facts could indicate that the effect of PPD is higher in early treatments because serum enzymes value gradually normalized and some symptoms disappeared, although the muscular weakness persisted for three days.

Furthermore, we studied how PPD-induced rhabdomyolysis affects central carbon metabolism in C2C12 muscle cell line. Thus, a first prospective approach applying metabolic Isotopomer Distribution Analysis (MIDA) showed significant different in metabolic pathway patterns when C2C12 cells are treated with PPD to induce rhabdomyolysis. More specifically, we found that glucose was metabolized to lactate mainly through glycolysis in both treated and non-treated C2C12 cells. However, glycolytic contribution to lactate production is higher in non-treated C2C12 cells. The low label incorporation observed in Glutamate suggest a reduced activity of TCA cycle in C2C12 cells, disruption of glyclolysis by PPD may provoke ATP depletion, leading to Ca+ accumulation related with rhabdomyolysis due to consequent low activity of ATP-depending Ca channel. On the other hand, LDH liberation after PPD treatment may explain glycolytic inhibiton, since LDH is needed to support glycolysis by recycling produced NADH to NAD^+^.

Additionally, differential changes in pentose phostphate pathways have been also observed. PPD cause a decrease in *de novo* synthesis of ribose-5-phosphate for nucleotide synthesis. The balance between non-oxidative and oxidative branches of pentose-phosphate pathway was also decreased in PPD treated cells. Since oxidative branch of pentose-phosphate pathway is used not only for pentose phosphate synthesis but also for the generation of reduction power as NADPH, this decrease may affect cellular detoxification machinery of Reactive Oxygen Species (ROS) (Dodson *et al*., 2013). Since ROS are well known to trigger apoptosis (Halliwell 2006), effects on ppp may correlate with effects on cell proliferation in vitro caused by these compounds, and specially, PPD inhibition of oxidative branch of ppp may explain enhanced apoptosis observed in treated cells (40% of apoptotic cells).

To further explore the metabolic effects of PPD-induced rhabdomyolysis we perform a metabolic flux analysis based kinetic modelling approach to analyze the tracer-based metabolic data of PPD-treated and control C2C12 cells. Our model-driven analysis permitted us to infer the metabolic flux alterations associated to PPD exposure. More specifically, our analysis suggest a higher glycolysis utilization compared with ppp activity in C2C12 cells which is in accordance with a higher enrichment of m2 lactate isotopologue (lactate generated from [1,2-^13^C_2_]-glucose via glycolysis) compared with m1 lactate isotopologue (lactate generated from [1,2-^13^C_2_]-glucose via glycolysis) in both cell groups. Furthermore, our model-driven analysis predicted a higher glycolytic activity relative to glucose consumption in control group that is consistent with the higher label incorporation observe in lactate in these cells. Our analysis also predicted a higher flux through the transketolase reaction that transforms fructorse-6-phosphate to xilulose5-phosphate in non-treated cells that is consistent with higher label enrichment in ribose-5-phosphate in control cells and with the MIDA analysis concluding a higher activity in the non-oxidative branch of ppp in control cells. Finally, the analysis determined a decrease in the flux through ATPase reaction leading to a reduction in ATP synthesis. Gathering these evidences we have an overview in which PPD reduces ribose and ATP production which is consistent with the reduction in cell viability observed in PPD-treated C2C12 cells an in Wistar Rats and is supported by the experimental evidences.

In conclusion, the present study demonstrates that PPD induces some pathologic signs involved in rhabdomyolysis such as muscle necrosis, release of muscle components into the circulation, myoglobinuria and muscle injury. Moreover, the present study has proven the potential of stable isotope techniques to understand the effects of PPD on central carbon metabolism. Our model-driven analysis has permitted to quantify the intracellular metabolic fluxes and the changes associated to PPD exposure which enabled a more in-depth understanding of the metabolic processes underlying the adverse effects associated with rhabdomyolysis on the C2C12 muscle cells. Thus, our work sheds new light on muscle dysfunction associated to PPD, opening new avenues for cost-effective therapies in Rhabdomyolysis disease.

**Author Contributions**

MMI: conception and design, assembly of data, data analysis, computational simulations and interpretation and manuscript writing. MS: collection and assembly of data, interpretation and manuscript writing. PG: collection and assembly of data, interpretation and manuscript writing. AA, RPJ, TR, and VP: Data interpretation and manuscript writing. SV: data analysis and interpretation and manuscript writing. LCA: Data interpretation and manuscript writing. CM.: conception and design, data analysis and interpretation, manuscript writing and final approval of manuscript. All authors read and approved the final version of the manuscript.

Conflict of Interest Statement

The authors declare that the research was conducted in the absence of any commercial or financial relationships that could be construed as a potential conflict of interest.

Supplementary Material

**Supplementary material 1**: Tables containing: i)Complet list of isotopologue fractions of Glucose, Glycogen, Lactate, Glutamate fraction C2-5 and C2-4 and Ribose-5-phosphate of treated and non-treated C2C12 cells, ii) Model-driven fitting of metabolite isotopologues and the corresponding χ² of treated and non-treated C2C12 cells, iii) kinetic parameters determined by our model-driven analysis and corresponding to the best fit solution, iv) absolute metabolic fluxes determined by our model-driven analysis and corresponding to the best fit solution and v) metabolic fluxes relative to hexokinase flux, determined by our model-driven analysis and corresponding to the best fit solution

**Supplementary material 2**: Detailed description of Isodyn software.

**Supplementary material 3**: Zip file with: i) the kinetic model used to determine the metabolic fluxes, ii) metabolic fluxes calculated for treated and non-treated C2C12 cells, iii) kinetic parameters defined for treated and non-treated C2C12 cells and iv) results of the experimental labeling data for treated and non-treated C2C12 cells

Acknowledgements

This work has received financial support from the government of Catalunya: ITT program of the Work Community of PyreneesAgència de Gestió d’Ajuts Universitaris i de Recerca (AGAUR) – Generalitat de Catalunya (2014SGR1017). CM acknowledges the support received through the prize “ICREA Academia” for excellence in research, funded by ICREA foundation – Generalitat de Catalunya. MMI and TR acknowledge the support received through the European Research Council under the European Union's Seventh Framework Programme (FP/2007–2013)/ERC Grant Agreement no. 320737.

**REFERENCES**

- Boren, J.; Lee, W. N.; Bassilian, S.; Centelles, J. J.; Lim, S.; Ahmed, S.; Boros, L. G.; Cascante, M. (2003). The stable isotope-based dynamic metabolic profile (SIDMAP) of butyrate induced HT29 cell differentiation. *J Biol Chem*. **278(31)**: 28395-402.
- Burchert A. (2007). Roots of imatinib resistance: a question of self-renewal? *Drug Resist Updat.* **10(4-5):**152-61.
- Castagné V, Wolinsky T, Quinn L, Virley D. (2012). Differential behavioral profiling of stimulant substances in the rat using the LABORAS™ system. *Pharmacol Biochem Behav.* **101(4):**553-63.
- Chavez LO, Leon M, Einav S, Varon J. (2016). Beyond muscle destruction: a systematic review of rhabdomyolysis for clinical practice. *Crit Care.* **20(1):**135.
- Chwaluk P. (2013). Rhabdomyolysis as an unspecyfic symptom of mushroom poisoning--a case report. *Przegl Lek.***70(8):**684-6.
- Cisternas P, Salazar P, Silva-Alvarez C, Barros LF, Inestrosa NC. (2016). Activation of Wnt signaling in cortical neurons enhances glucose utilization through glycolysis. *J Biol Chem.* **pii**: jbc.M116.735373.
- Cortés R, Crespo M, Davin L, Martín R, Quirante J, Ruiz D, Messeguer R, Calvis C, Baldomà L, Badia J, Font-Bardía M, Calvet T, Cascante M. (2012). Seven-membered cycloplatinated complexes as a new family of anticancer agents. X-ray characterization and preliminary biological studies. *Eur J Med Chem.* **54**:557-66.
- Cowan-Jacob SW, Guez V, Fendrich G, Griffin JD, Fabbro D, Furet P, Liebetanz J, Mestan J, Manley PW. (2004). Imatinib (STI571) resistance in chronic myelogenous leukemia: molecular basis of the underlying mechanisms and potential strategies for treatment. *Mini Rev Med Chem.* **4(3):**285-99.
- Dodson M, Darley-Usmar V, Zhang J. (2013). Cellular metabolic and autophagic pathways: traffic control by redox signaling. *Free Radic Biol Med.* **63**:207-21.
- Fajardo VA, McMeekin L, Basic A, Lamb GD, Murphy RM, LeBlanc PJ. (2013). Isolation of sarcolemmal plasma membranes by mechanically skinning rat skeletal muscle fibers for phospholipid analysis. *Lipids*. **8(4)**:421-30.
- Garrigue JL, Ballantyne M, Kumaravel T, Lloyd M, Nohynek GJ, Kirkland D, Toutain H. (2006). In vitro genotoxicity of para-phenylenediamine and its N-monoacetyl or N,N'-diacetyl metabolites. *Mutat Res*. **608(1):**58-71.
- Halliwell, B. (2006). Reactive species and antioxidants. Redox biology is a fundamental theme of aerobic life. *Plant Physiol.* **141**:312-322.
- Hooff GP, van Huizen NA, Meesters RJ, Zijlstra EE, Abdelraheem M, Abdelraheem W, Hamdouk M, Lindemans J, Luider TM. (2011). Analytical investigations of toxic p-phenylenediamine (PPD) levels in clinical urine samples with special focus on MALDI-MS/MS. *PLoS One*. **6(8):**e22191.
- Hortelano Araque A, Fernández Calderón C, Ureta Tolsada MP, Uña Orejón R. (2008). habdomyolysis and acute renal failure. *Rev Esp Anestesiol Reanim.* **55(4)**:261.
- Iannotti FA, Panza E, Barrese V, Viggiano D, Soldovieri MV, Taglialatela M. (2010). Expression, localization, and pharmacological role of Kv7 potassium channels in skeletal muscle proliferation, differentiation, and survival after myotoxic insults. *J Pharmacol Exp Ther.* **332(3):**811-20.
- Kominsky DJ, Klawitter J, Brown JL, Boros LG, Melo JV, Eckhardt SG, Serkova NJ. (2009). Abnormalities in glucose uptake and metabolism in imatinib-resistant human BCR-ABL-positive cells. *Clin Cancer Res.* **15(10):**3442-50.
- Mao CS, Bassilian S, Lim SK, Lee WN. (2002). Underestimation of gluconeogenesis by the [U-(13)C(6)]glucose method: effect of lack of isotope equilibrium. *Am J Physiol Endocrinol Metab.* **282(2):**E376-85.
- Marin de Mas I, Selivanov VA, Marin S, Roca J, Orešič M, Agius L, Cascante M. (2011). Compartmentation of glycogen metabolism revealed from 13C isotopologue distributions. *BMC Syst Biol.* **5**:175.
- Melli G, Chaudhry V, Cornblath DR. (2005). Rhabdomyolysis: an evaluation of 475 hospitalized patients. *Medicine (Baltimore).* **84(6):**377-85.
- Pachon, G.; Rasoanaivo, H.; Azqueta, A.; Rakotozafy, J. C.; Raharisololalao, A.; De Cerain, A. L.; De Lapuente, J.; Borras, M.; Moukha, S., Centelles, J. J.; Creppy, E. E.; Cascante, M. (2007). Anticancer effect of a new benzophenanthridine isolated from Zanthoxylum madagascariense (Rutaceline).*In Vivo* **21(2):**417-22.
- Pachón, O. G.; Azqueta A.; Lavaggi, M. L.; López de Cerain, A.; Creppy, E. E.; Collins, A.; Cerecetto, H.; González, M.; Centelles, J. J. and Cascante, M. (2008). Antitumoral Effect of Phenazine N5,N10-Dioxide Derivatives on Caco-2 Cells. *Chem. Res. Toxicol*. **21(8)**:1578-85.
- Pourahmad J, O'Brien PJ, Chan K, Shakouri A. (2008). Tetramethylphenylenediamine-induced hepatocyte cytotoxicity caused by lysosomal labilisation and redox cycling with oxygen activation*. Chem Biol Interact.* **172(1):**39-47.
- Selivanov, V. A; Puigjaner, J.; Sillero, A.; Centelles, J. J.; Ramos-Montoya, A.; Lee, P. W. and Cascante, M. (2004). An Optimized Algorithm for Flux Estimation from Isotopomer Distribution in Glucose Metabolites. *Bioinformatics* **20(18):**3387-97.
- Selivanov, V. A.; Meshalkina, L. E.; Solovjeva, O. N.; Kuchel, P. W.; Ramos-Montoya, A.; Kochetov, G. A.; Lee, P. W.; Cascante, M. (2005). Rapid simulation and analysis of isotopomer distributions using constraints based on enzyme mechanisms: an example from HT29 cancer cells. *Bioinformatics*. **21(17):**3558-64.
- Selivanov, V. A.; Sukhomlin, T.; Centelles, J. J.; Lee, P. W.; Cascante, M. (2006). Integration of enzyme kinetic models and isotopomer distribution analysis for studies of in situ cell operation. *BMC Neurosci*. **7** Suppl 1:S7.
- Soni SS, Nagarik AP, Dinaker M, Adikey GK, Raman A. (2009). Systemic toxicity of paraphenylenediamine. *Indian J Med Sci.* **63(4):**164-6.
- Vizan, P.; Boros, L. G.; Figueras, A.; Capella, G.; Mangues, R.; Bassilian, S.; Lim, S.; Lee, W. N.; Cascante M. (2005). K-ras codon-specific mutations produce distinctive metabolic phenotypes in mice fibroblasts. *Cancer Res.* **65(13):** 5512-5.
- Wamelink MM, Struys EA, Jakobs C. (2008). The biochemistry, metabolism and inherited defects of the pentose phosphate pathway: a review. *J Inherit Metab Dis.* **31(6):**703-17.
- Wilson S. (2006). Rhabdomyolysis made easy. *Aust Nurs J*. **14(2):**21-3.
- Yoo H, Antoniewicz MR, Stephanopoulos G, Kelleher JK. (2008). Quantifying reductive carboxylation flux of glutamine to lipid in a brown adipocyte cell line. *J Biol Chem.* **283(30):**20621-7.
- Zoll J, Bouitbir J, Sirvent P, Klein A, Charton A, Jimenez L, Péronnet FR, Geny B, Richard R. (2015). Apparent Km of mitochondria for oxygen computed from Vmax measured in permeabilized muscle fibers is lower in water enriched in oxygen by electrolysis than injection. *Drug Des Devel Ther.* **9**:3589-97.
